# Therapeutic Galectin‐3 Apheresis Improves Sepsis Outcomes Through Coordinated Neutrophil Modulation and Endothelial Barrier Preservation: A Translational Study

**DOI:** 10.1002/mco2.70659

**Published:** 2026-03-15

**Authors:** Zhongyi Sun, Jiachen Qu, Sheng Peng, Yanan Hu, Amity Eliaz, Glenn M. Chertow, Isaac Eliaz, Zhiyong Peng

**Affiliations:** ^1^ Department of Critical Care Medicine Zhongnan Hospital of Wuhan University Wuhan Hubei China; ^2^ Clinical Research Center of Hubei Critical Care Medicine Wuhan Hubei China; ^3^ Department of Neurology University of California San Francisco California USA; ^4^ Department of Medicine Epidemiology and Population Health, and Health Policy Stanford University School of Medicine Stanford California USA; ^5^ Amitabha Medical Clinic and Healing Center Santa Rosa California USA

**Keywords:** extracorporeal therapy, Galectin‐3, organ dysfunction, sepsis, survival

## Abstract

Sepsis remains a leading cause of global mortality, characterized by uncontrolled inflammation and multi‐organ dysfunction. Galectin‐3 (Gal‐3) is a damage‐associated molecular pattern (DAMP) protein that amplifies inflammatory cascades during sepsis and represents a potential therapeutic target. We conducted an integrated translational investigation combining clinical observation (87 septic patients, 27 healthy volunteers) with preclinical Gal‐3 removal using an anti‐Gal‐3 apheresis column in two sepsis models: a rat cecal ligation and puncture (CLP) model (*n *= 48) and a porcine lipopolysaccharide (LPS)‐induced model (*n* = 31). Mechanistic assessments included serum testing, multi‐omics profiling, invasive hemodynamic monitoring, and histopathology. Patients with sepsis exhibited markedly elevated Gal‐3 levels (*p* < 0.001), and survivors showed progressive Gal‐3 decline compared with non‐survivors (*p* < 0.01). Gal‐3 removal significantly improved survival in rats (57.1% vs. 25.0%, *p* = 0.003) and pigs (68.8% vs. 26.7%, *p* = 0.004). Treatment attenuated neutrophil activation and tissue infiltration, preserved endothelial barrier integrity, and modulated pro‐survival and hypoxia‐response signaling pathways, accompanied by reduced vasopressor requirements and pulmonary edema. Collectively, these findings demonstrate that Gal‐3 removal improves survival and reduces organ damage in preclinical sepsis models in association with coordinated neutrophil modulation and endothelial barrier preservation, highlighting Gal‐3 as a promising therapeutic target in sepsis.

## Graphical abstract

Gal‐3 apheresis reduces neutrophil hyperactivation (CXCL2/CXCL8, MPO, NETs), preserves endothelial barrier function (tight junctions, vWF/VCAM‐1/ICAM‐1), and attenuates PI3K/AKT/HIF‐1α signaling. These coordinated effects significantly improve hemodynamics, reduce pulmonary edema (ELWI), lower vasopressor and fluid requirements, and increase survival, highlighting Gal‐3 as a therapeutic target in sepsis

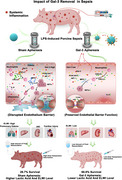

## 1 Introduction

Sepsis presents one of the most formidable clinical challenges, affecting millions of patients worldwide with mortality rates that have shown limited improvement despite decades of research [1, 2]. The fundamental difficulty in sepsis treatment stems from the delicate balance between beneficial and harmful host responses. This duality has complicated therapeutic development, as interventions must preserve protective immunity while mitigating harmful inflammation [2, 3]. Inflammatory mechanisms essential for pathogen clearance can become dysregulated and contribute to tissue injury and organ failure [1, 2, 3, 4]. Current treatment approaches remain largely supportive, and the interventions targeting the underlying pathophysiology of sepsis are lacking [3, 5].

Numerous clinical trials have evaluated targeted interventions for sepsis, including anti‐inflammatory agents, immunomodulators, and antioxidants, with limited efficacy [1, 2, 5, 6]. These results have highlighted the complexity of sepsis pathophysiology. Recent studies suggest that more effective therapeutic strategies may require intervention at central regulatory points that coordinate multiple pathophysiological processes [3, 7].

Galectin‐3 (Gal‐3), a β‐galactoside‐binding lectin, has emerged as a potential therapeutic target based on its established involvement in inflammatory processes [8, 9]. Gal‐3 is rapidly upregulated during sepsis and has been shown to correlate with adverse outcomes in clinical studies [10, 11, 12]. Studies have shown that Gal‐3 upregulation precedes elevations in Interleukin‐6 (IL‐6), highlighting Gal‐3 as an upstream mediator of the inflammatory cascade [13, 14, 15, 16]. In addition, recent evidence suggests that inhibition or genetic deletion of Gal‐3 can provide protection against sepsis‐induced organ injury [14, 17]. However, it is difficult for inhibition or genetic deletion to be clinically translated. In addition, the specific mechanisms through which Gal‐3 influences sepsis pathophysiology remain incompletely characterized.

Therapeutic apheresis offers a viable approach for removal of specific mediators from the circulation [18]. While broad‐spectrum cytokine removal has shown mixed results in sepsis trials [19, 20], more targeted approaches may offer improved therapeutic outcomes [21]. Prior studies have demonstrated that selective extracorporeal removal of Gal‐3 is both feasible and biologically active in vivo. In a porcine Complete Freund Adjuvant (CFA) induced skin inflammation model, Gal‐3 apheresis effectively depleted circulating Gal‐3, resulting in significantly faster resolution of skin erythema and restoration of normal epithelial integrity compared with sham apheresis [22, 23]. These findings suggested that the removal of a key regulatory molecule like Gal‐3 could potentially modulate multiple pathways while minimizing off‐target effects.

Based on this rationale, Gal‐3 apheresis was evaluated for its therapeutic potential in two well‐characterized animal models of sepsis: a rat model of cecal ligation and puncture (CLP)‐induced sepsis, and a porcine model of lipopolysaccharide (LPS)‐induced sepsis. Our objectives were to assess the impact of Gal‐3 removal on sepsis outcomes and to characterize the underlying mechanisms through comprehensive physiological, biochemical, and histopathological analyses.

## 2 Results

### 2.1 Serum Gal‐3 Levels Are Higher in Patients With Sepsis and Downtrending Levels Are Associated With 28‐Day Survival

**FIGURE 1:**
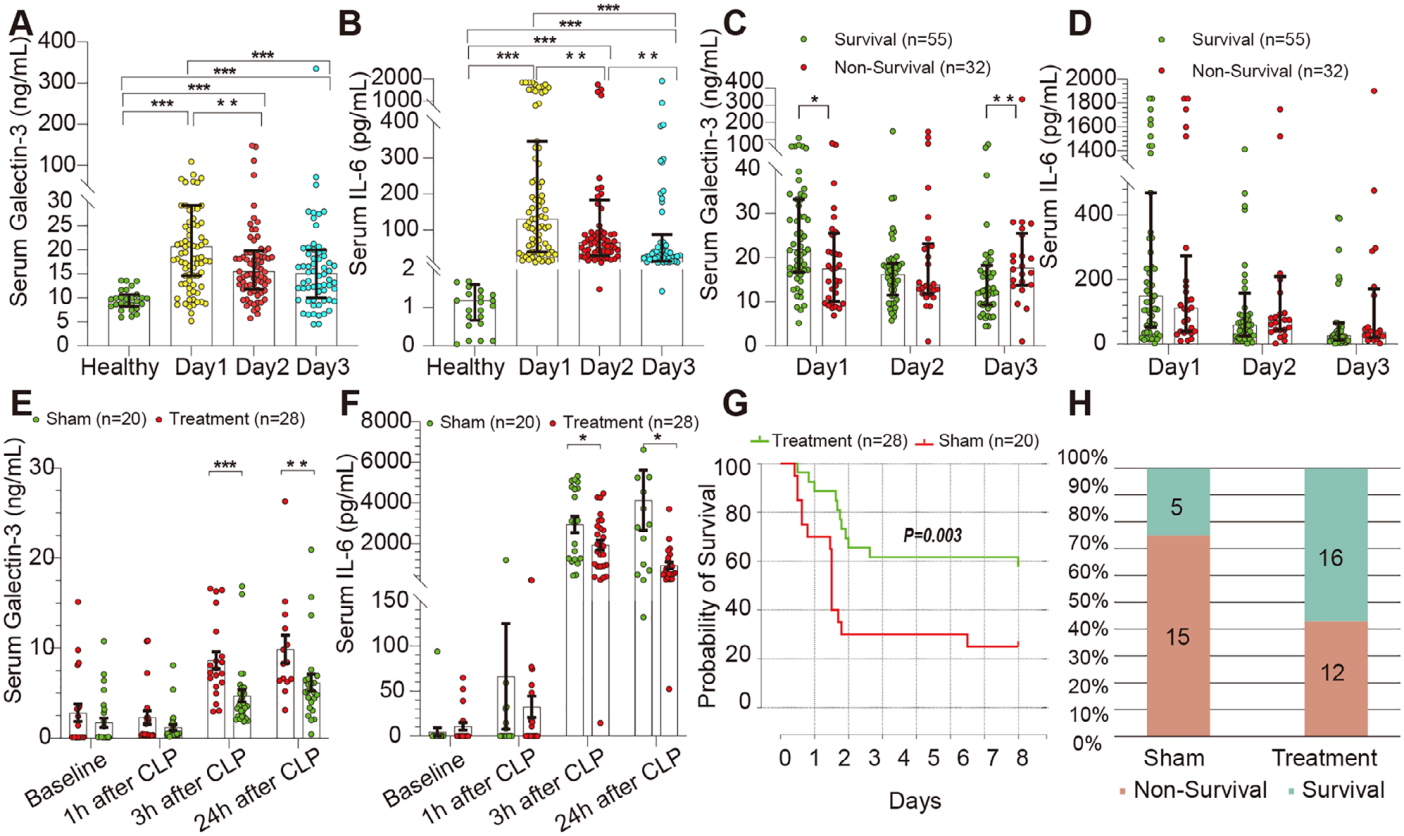
Divergent temporal dynamics of circulating Galectin‐3 and IL‐6 in human sepsis and therapeutic effects of Gal‐3 depletion in a rat CLP model. (A)–(B) Serum Gal‐3 and IL‐6 concentrations in healthy volunteers and patients with sepsis were measured on Days 1, 2, and 3 after diagnosis. (C)–(D) Serum Gal‐3 and IL‐6 levels in patients with sepsis stratified by 28‐day survival status at each indicated time point. (E)–(F) Serum Gal‐3 and IL‐6 concentrations in rats subjected to CLP, measured at baseline and at 1, 3, and 24 h after CLP in sham and Gal‐3 depletion treatment groups. (G) Kaplan‐Meier survival curves of CLP‐induced septic rats over a 7‐day observation period. (H) Distribution of survival and non‐survival outcomes at Day 7 in sham and Gal‐3 depletion‐treated rats. Serum Gal‐3 and IL‐6 levels were quantified by ELISA. Data are presented as individual values with bars indicating mean ± SEM. All statistical comparisons were performed at predefined time points using nonparametric two‐group comparisons. Differences between groups were analyzed using the Mann‐Whitney U test. Survival curves were analyzed using the log‐rank test.*p < 0.05, **p < 0.01, ***p < 0.001.

We enrolled 87 consecutive sepsis patients and 27 healthy controls (Table S1). Gal‐3 levels were significantly higher in patients with sepsis than in healthy individuals (median, 20.7 vs. 9.7 ng/mL), corresponding to a median difference of 11.0 ng/mL (95% CI, 8.57–13.41; *p* < 0.001; Cliff's *δ* = 0.79). IL‐6 levels showed an even greater divergence (median, 130.9 vs. 1.2 pg/mL), with a median difference of 129.70 pg/mL (95% CI, 103.40–182.67; *p* < 0.001; Cliff's δ = 1.00). Both biomarkers remained consistently higher in patients with sepsis than in healthy controls across the 72‐h observation period (Figure 1A–B). To explore the relationship between biomarker trajectories and survival, we stratified patients by 28‐day survival. Baseline characteristics, including age, APACHE II score, SOFA score, and serum lactate, were well‐balanced between survivors (*n* = 55) and non‐survivors (*n* =  32) (all *p* > 0.05; Table S2), ensuring that subsequent biomarker differences would not be confounded by baseline disease severity.

Notably, Gal‐3 trajectories diverged significantly by survival outcome. Survivors exhibited progressive declines in Gal‐3 levels, reaching significantly lower levels by Day 3 compared with non‐survivors (median, 12.4 vs. 18.1 ng/mL; *p* = 0.007; rank‐biserial *r* = 0.42; 95% CI, 0.15–0.67; medium effect) (Figure 1C). In contrast, IL‐6 dynamics did not significantly differ between groups at any timepoint, downtrending similarly in both survivors and non‐survivors (Day 3: *p* = 0.37; rank‐biserial *r* = 0.14) (Figure 1D).

### 2.2 Gal‐3 Apheresis in CLP‐Induced Polymicrobial Sepsis Is Associated With Lower Serum IL‐6 and Higher 7‐Day Survival

Given the clinical observation of Gal‐3 clearance kinetics in clinical sepsis, we investigated whether therapeutic Gal‐3 depletion could improve outcomes in experimental sepsis. We performed whole blood apheresis in rats with polymicrobial sepsis. Treatment and sham groups showed comparable baseline and 1‐h post‐CLP serum Gal‐3 levels [0.5 (0.4, 1.5) vs. 0.5 (0.3, 2.3) ng/mL, *p* > 0.05]. Following 2‐h apheresis initiated at 1 h post‐CLP, treated animals exhibited sustained Gal‐3 reduction at 3 h [3.8 (2.4, 5.6) vs. 7.5 (5.2, 12.6) ng/mL, *p* < 0.001] and 24 h post‐CLP [5.2 (3.5, 6.5) vs. 8.4 (6.4, 12.6) ng/mL, *p* < 0.01, Figure 1E]. Gal‐3 depletion significantly attenuated systemic inflammation, with reduced serum IL‐6 at 3 h [1812.2 (880.1, 2817.5) pg/mL vs. 3056.9 (1229.1, 4765.6) pg/mL, *p* < 0.05] and 24 h post‐CLP [646.1 (463.4, 1259.2) vs. 2861.0 (619.4, 5252.1) pg/mL, *p* < 0.05, Figure 1F]. Gal‐3 apheresis was well tolerated in rats with no procedure‐related complications or hemodynamic instability observed. Gal‐3 apheresis significantly improved 7‐day survival: 57.1% (16/28) of treated rats survived versus 25% (5/20) of sham rats (log‐rank *p* = 0.003, Figure 1G,H). These findings demonstrated that Gal‐3 removal attenuated systemic inflammation and enhanced survival in a clinically‐relevant animal model.

### 2.3 Sepsis Model Validation and Baseline Characteristics

To validate these findings in a large animal model, we evaluated Gal‐3 apheresis in pigs with endotoxin‐induced sepsis. Of 34 instrumented pigs, 3 were excluded before randomization because of anesthesia‐related complications unrelated to treatment allocation. The remaining 31 were randomized to Gal‐3 apheresis (*n* = 16) or sham apheresis (*n* = 15). There were no post‐randomization exclusions. Time‐point missingness (e.g., temporary line obstruction or equipment downtime) was handled within mixed‐effects models without imputation. Model validation confirmed development of severe sepsis characterized by >30% reduction in mean arterial pressure (MAP), heart rate >120 beats per minute, and elevated lactate levels within 2 h of LPS initiation in both sham and treatment groups (Table S3).

### 2.4 Gal‐3 Apheresis Is Associated With Sustained Gal‐3 Reduction, Lower IL‐6 Levels, Reduced Vasopressor and Fluid Requirements, and Higher 24‐h Survival

**FIGURE 2:**
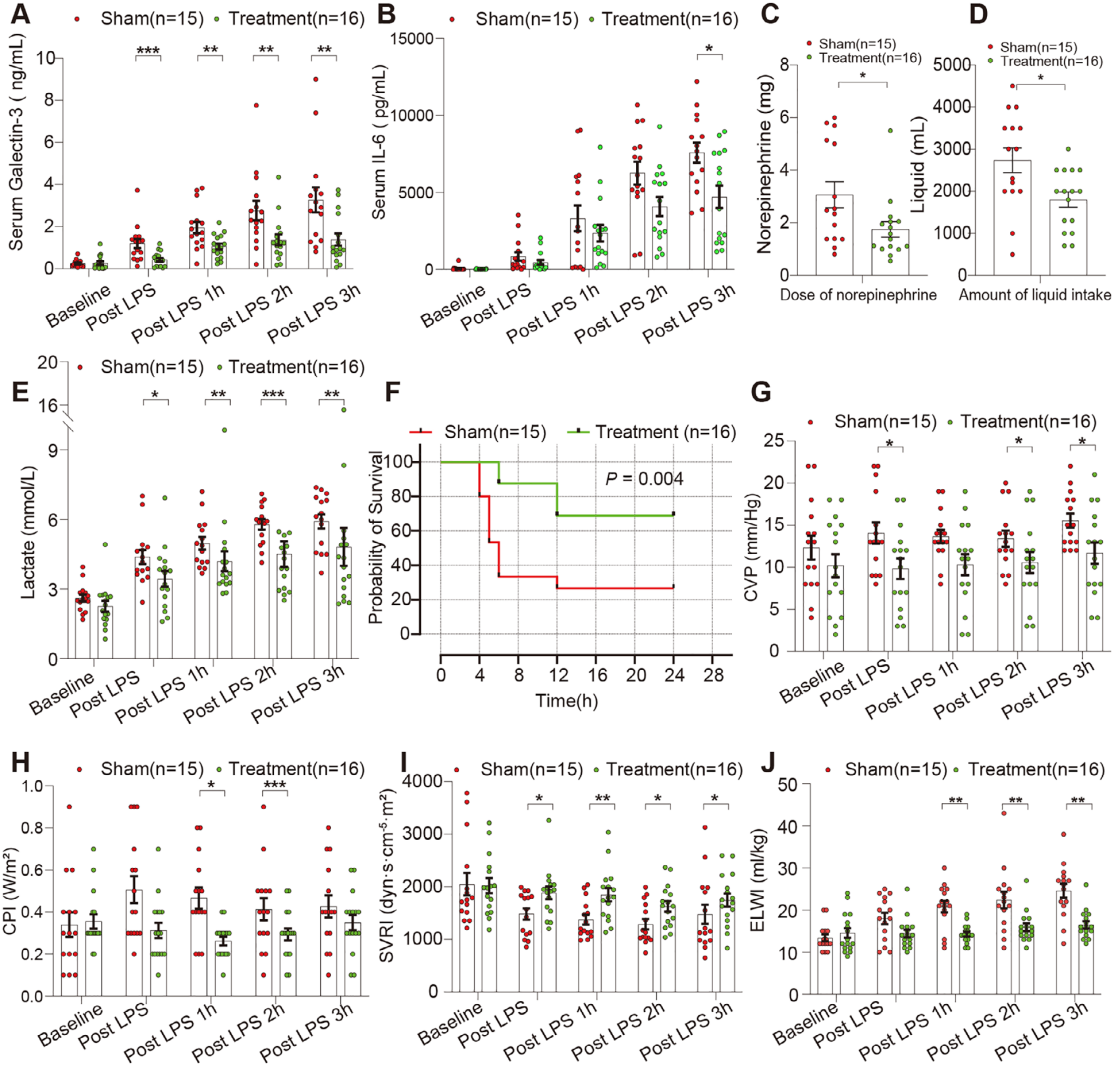
Galectin‐3 apheresis is associated with reduced systemic inflammation, improved hemodynamics, and enhanced short‐term survival in a porcine sepsis model. (A) Serum Gal‐3 concentrations measured at baseline and at indicated time points following LPS challenge in sham and Gal‐3 apheresis–treated pigs. (B) Longitudinal changes in serum IL‐6 concentrations after LPS administration in sham and treatment groups. (C) Cumulative norepinephrine dose administered to maintain the target mean arterial pressure throughout the study. (D) Total volume of intravenous fluid administered during resuscitation. (E) Blood lactate concentrations measured over time during the experimental period. (F) Kaplan‐Meier survival curves over a 24‐h observation period for sham and Gal‐3 removal‐treated pigs. (G) CVP measured at predefined time points during hemodynamic monitoring. (H) CPI assessed over time as an index of global cardiac performance. (I) SVRI measured longitudinally to assess systemic vascular tone. (J) ELWI measured at predefined time points as an indicator of pulmonary fluid accumulation. Data are presented as individual values with bars indicating mean ± SEM. All statistical comparisons were performed at predefined time points using nonparametric two‐group comparisons (Mann‐Whitney U test). Survival differences were analyzed using the log‐rank test. *p < 0.05, **p < 0.01, ***p < 0.001.

The apheresis system achieved consistent Gal‐3 clearance throughout treatment. Circulating Gal‐3 levels were significantly reduced in the treatment group as early as 35 min post‐apheresis initiation compared with sham [0.4 (0.1–0.6) vs. 1.3 (0.5–1.5) ng/mL, *p* = 0.001], and this reduction persisted through all subsequent time points [1 h: 1.0 (0.6–1.5) vs. 1.7 (1.2–2.3) ng/mL; 2 h: 1.2 (0.7–1.6) vs. 2.3 (1.7–3.5) ng/mL; 3 h: 0.9 (0.7–2.4) vs. 2.9 (1.3–3.8) ng/mL; all *p* < 0.01] (Figure 2A). This sustained Gal‐3 reduction was accompanied by significant attenuation of the systemic inflammatory response. IL‐6 concentrations at 3 h post‐LPS were markedly lower in treated animals compared to sham [4767.3 (1805.0–7551.7) vs. 7672.7 (5699.5–9704.6) pg/mL, *p* < 0.05] (Figure 2B). Norepinephrine requirements were significantly reduced [1.5 (1.0–2.0) vs. 2.5 (1.4–5.1) mg total dose, *p* < 0.01], as were cumulative fluid requirements [2000 (1075–2500) vs. 3000 (2000–3500) mL, *p* < 0.05], indicating reduced capillary leak and preservation of vascular integrity, as well as improved hemodynamic stability (Figure 2C–D). Gal‐3 apheresis was associated with improved lactate kinetics (4.8 ± 3.3 vs. 5.9 ± 1.2 mmol/L, *p* < 0.05), indicating preserved cellular energetics and tissue perfusion (Figure 2E). Notably, 24‐h survival was significantly higher in the treatment group (68.8% vs. 26.7%, *p* = 0.004, Figure 2F).

Gal‐3 apheresis was technically feasible and well tolerated in the porcine model. No device‐related adverse events, circuit malfunction, or catheter complications occurred. Hemodynamic parameters, including MAP and heart rate, remained within the expected range for endotoxemic animals and showed no procedure‐associated instability (Table S3, Table S6). Biochemical indices of organ injury (creatinine, aspartate aminotransferase, alanine aminotransferase) remained stable over time without evidence of treatment‐related toxicity (Table S4). Pulmonary mechanics and routine hematologic values similarly demonstrated no adverse changes attributable to apheresis (Tables S5–S7). These findings demonstrated that Gal‐3 apheresis did not introduce detectable safety concerns and was operationally feasible throughout the intervention period.

### 2.5 Gal‐3 Apheresis Is Associated With Improved Cardiac Function, Vascular Resistance, and Extravascular Lung Water

Central venous pressure (CVP) analysis revealed markedly different patterns between groups. While baseline CVP was comparable, sham animals showed progressive elevation reaching 15.5 ± 3.3 mmHg at 3 h, whereas treated animals maintained stable values (11.7 ± 5.1 mmHg at 3 h), reflecting superior cardiac preload management (Table S6, Figure 2G). Cardiac function was better preserved in treated animals, with cardiac power index remaining stable (0.4 ±0.1 to 0.4 ±0.2 W/m^2^) compared to progressive dysfunction in the sham group (Table S6, Figure 2H). Systemic vascular resistance index(SVRI) analysis revealed striking preservation of vascular function in treated animals. Sham animals experienced 21% SVRI decline, while treated animals showed only a modest 14% decrease, reflecting preserved vascular responsiveness (Table S6, Figure 2I).

Hemodynamic and pulmonary assessments demonstrated marked protection against acute lung injury. Sham animals developed severe pulmonary edema, with extravascular lung water index (ELWI) increasing 84% (13.4 ± 3.2 to 24.6 ± 6.4 mL/kg), whereas treated animals exhibited only a 13% increase (from 14.6 ± 4.7 to 16.5 ± 3.4 mL/kg), consistent with preserved alveolar capillary barrier integrity (Table S6, Figure 2J).

### 2.6 Gal‐3 Apheresis Reduces Organ Injury and Preserves Tissue Architecture

**FIGURE 3:**
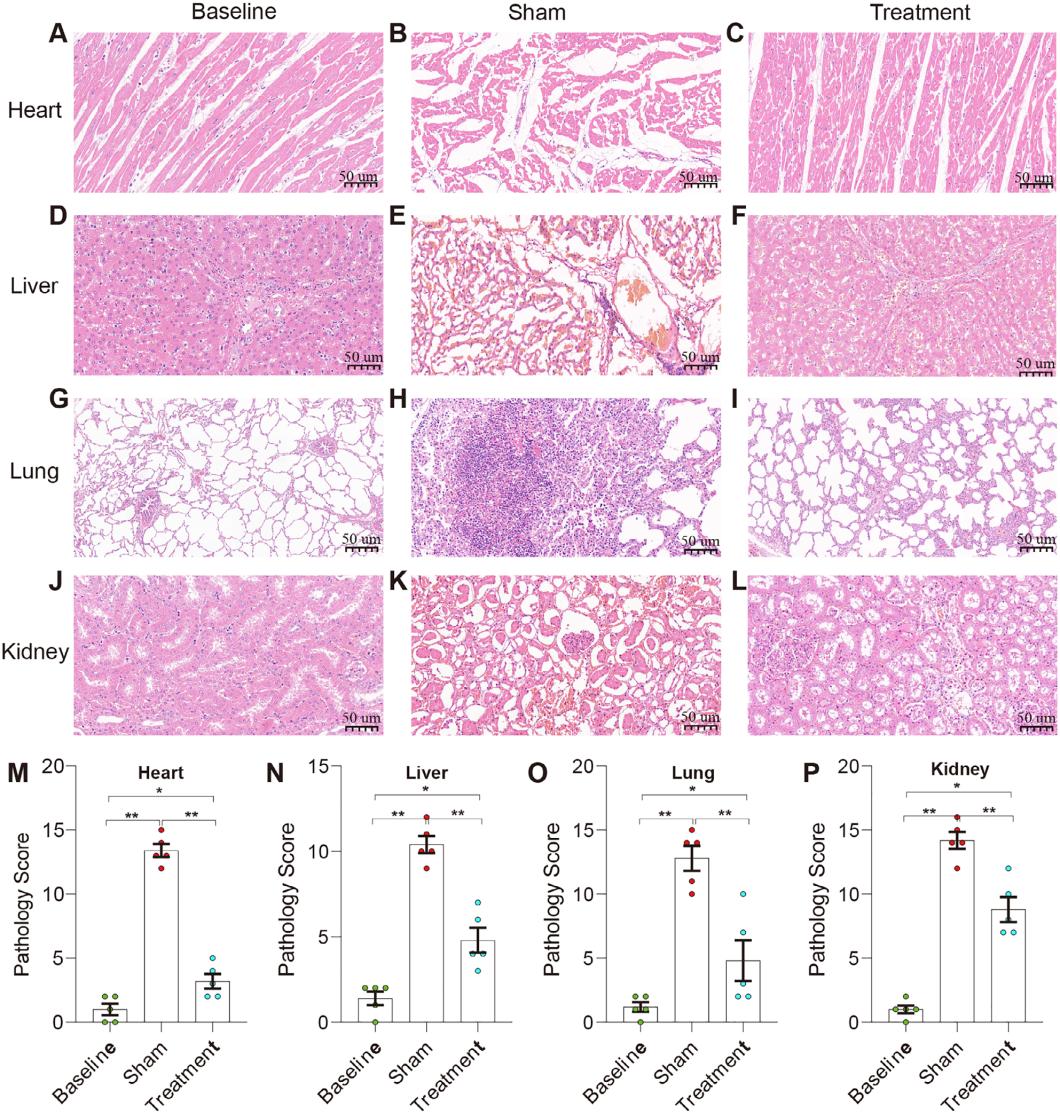
(A)–(L) Representative H&E‐stained sections of heart (A)–(C), liver (D)–(F), lung (G)–(I), and kidney (J)–(L) collected at 24 h from baseline, sham, and treatment groups. Scale bar: 50 µm. (M)–(P) Semiquantitative injury scores for heart (M; 0–16), liver (N; 0–16), lung (O; 0–20), and kidney (P; 0–16) were assigned by a pathologist blinded to group allocation using prespecified, organ‐specific criteria (see Methods). Lung scores were calculated as the sum of 5 domains (0‐4 each; total 0‐20), and heart/liver/kidney scores as the sum of 4 domains (0–4 each; total 0–16). For each animal, 5 randomly selected high‐power fields per organ were scored using standardized sampling sites, and values were averaged to yield one score per organ per animal. Data are shown as individual values with mean ± SEM. Prespecified pairwise comparisons were performed using two‐tailed Mann‐Whitney U tests. *p < 0.05, **p < 0.01, ***p < 0.001.

Histopathological examination of tissues from surviving animals at 24 h revealed extensive organ protection. Cardiac tissue from the treatment group showed preserved cardiomyocyte morphology and intercalated disc integrity, contrasting with the cellular disruption and inflammatory infiltration observed in the sham group. Pulmonary tissue demonstrated maintained alveolar architecture with minimal inflammatory changes, compared to the diffuse alveolar damage and neutrophil infiltration evident in sham. Hepatic examination revealed preserved lobular architecture and minimal hepatocellular injury in treated animals, while sham animals showed extensive necrosis and inflammatory infiltration. Renal tissue similarly demonstrated reduced injury, with maintained glomerular morphology and tubular integrity in the treatment group versus the acute tubular necrosis and interstitial inflammation observed in the sham group. Quantitative injury scoring confirmed significant protection across all examined organs (Figure 3A–P).

### 2.7 Gal‐3 Apheresis Induces Coordinated Endothelial‐Immune‐Metabolic Reprogramming in Sepsis

**FIGURE 4:**
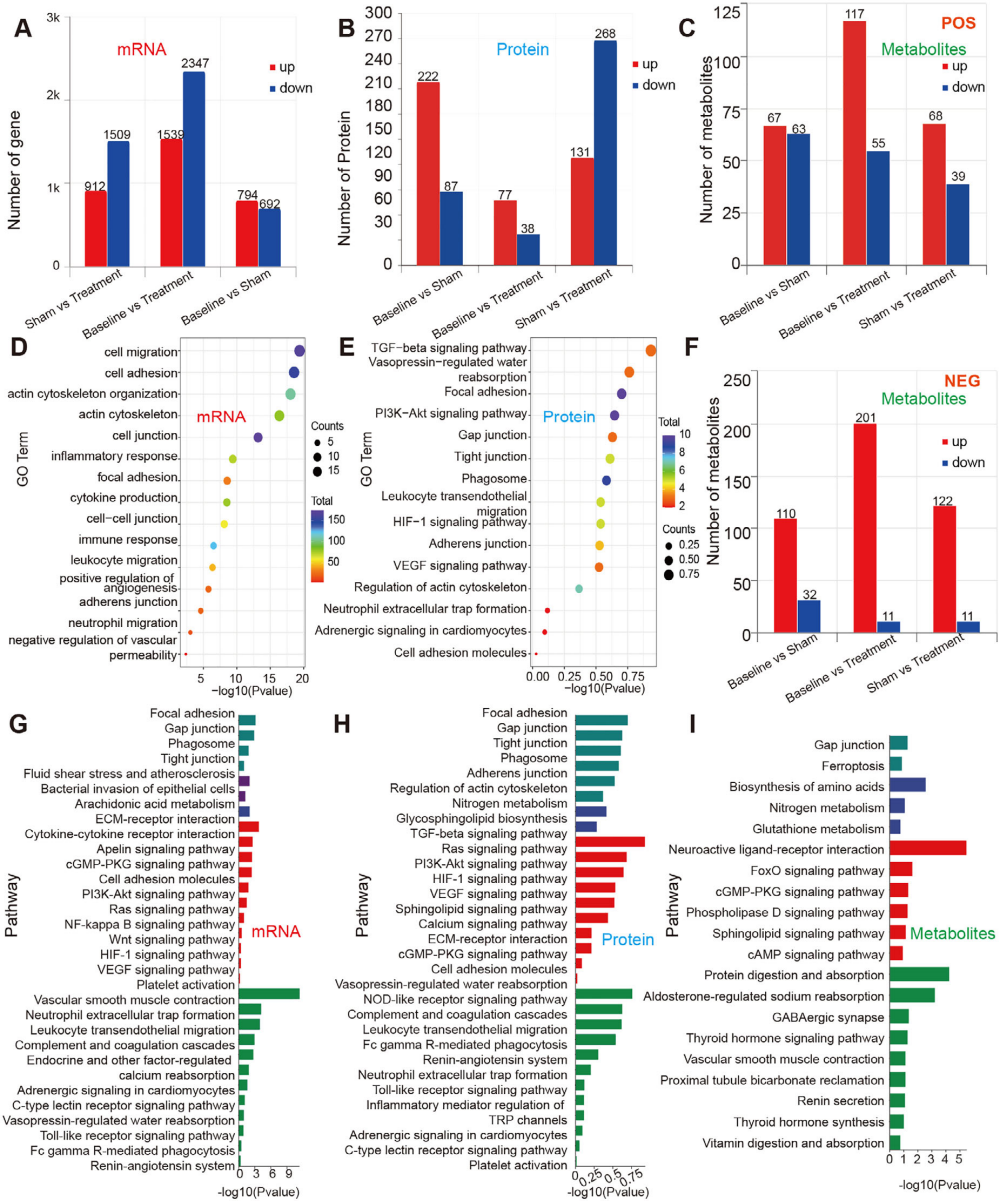
Multi‐omics features associated with Galectin‐3 adsorption in a porcine sepsis model. (A) Numbers of differentially expressed transcripts (upregulated and downregulated) across the indicated pairwise comparisons (sham vs. treatment, baseline vs. treatment, and baseline vs. sham). Differential expression was assessed using DESeq2, with significance defined as |log2(fold change)| > 1 and *p* < 0.05. (B) Numbers of differentially abundant proteins (upregulated and downregulated) across the indicated pairwise comparisons, defined by |log2(fold change)| > 1 and *p* < 0.05. (C) Numbers of differential metabolites detected in positive ion mode (POS) across the indicated pairwise comparisons. Differential metabolites were defined using OPLS‐DA (VIP > 1) and two‐tailed Student's *t* test (*p* < 0.05). (D)–(E) GO enrichment analysis of differentially expressed transcripts (D) and differentially abundant proteins (E) comparing sham versus treatment. Dot size indicates the number of mapped features (Counts) assigned to each term, and color indicates enrichment significance (–log10 *P*). Counts are not intended for direct comparison across omics layers because the number of significant features and annotation coverage differ by dataset. (F) Numbers of differential metabolites detected in negative ion mode (NEG) across the indicated pairwise comparisons, defined by OPLS‐DA (VIP > 1) and two‐tailed Student's *t*‐test (*p* < 0.05). (G)–(I) KEGG pathway enrichment analysis of differential transcripts (G), proteins (H), and metabolites (I) comparing sham versus treatment. Bar length indicates enrichment significance (–log10 *P*). Bar colors denote KEGG BRITE Level 1 functional categories: teal, Cellular Processes; purple, Human Diseases; red, Environmental Information Processing; dark blue, Metabolism; green, Organismal Systems.

Integrated transcriptomic, proteomic, and metabolomic analyses across baseline, sham, and treatment groups revealed a treatment‐associated state transition. Transcriptomic remodeling was greatest in baseline versus treatment (3886 differentially expressed genes (DEGs); 1539 upregulated and 2347 downregulated), compared with baseline versus sham (1486 DEGs) and sham versus treatment (2421 DEGs) (Figure 4A). Proteomic changes were directionally biased in sham versus treatment (399 differentially expressed proteins [DEPs]), whereas baseline versus treatment showed a smaller proteomic footprint (115 DEPs) (Figure 4B). Metabolomics in both ion modes further supported systemic reconfiguration, including enrichment of redox and stress‐adaptation pathways, such as glutathione metabolism and ferroptosis‐associated programs (Figure 4C,F,I). These findings highlighted oxidative stress handling as a metabolic correlate of treatment. Supporting principal component analysis (PCA), volcano plots, and overlap analyses are shown in Figure S1–S4.

Gene Ontology (GO) and Kyoto Encyclopedia of Genes and Genomes (KEGG) analyses consistently demonstrated endothelial junction/adhesion and cytoskeletal organization (focal adhesion; adherens/tight/gap junction; regulation of actin cytoskeleton), together with pathways governing leukocyte‐endothelial interactions (leukocyte transendothelial migration; cell adhesion molecules) and immunothrombotic circuitry (platelet activation; complement and coagulation cascades) (Figure 4D,E,G,H). Nucleotide‐binding oligomerization domain (NOD)‐like receptor (NLR) and neutrophil extracellular trap (NET) formation (NETosis)‐related signatures were consistent with modulation of excessive neutrophil activation and reduced endothelial injury potential (Figure 4H). In addition, engagement of phosphoinositide 3‐kinase (PI3K)‐AKT (protein kinase B), vascular endothelial growth factor (VEGF), and hypoxia‐inducible factor 1 (HIF‐1) signaling linked barrier stabilization to survival/repair and vascular tone mechanotransduction pathways (Figure 4G,H). Together, these profiles provide a mechanistic scaffold connecting Gal‐3 removal to coordinated modulation of the neutrophil‐endothelial interface, reducing excess activation and preserving barrier function.

**FIGURE 5:**
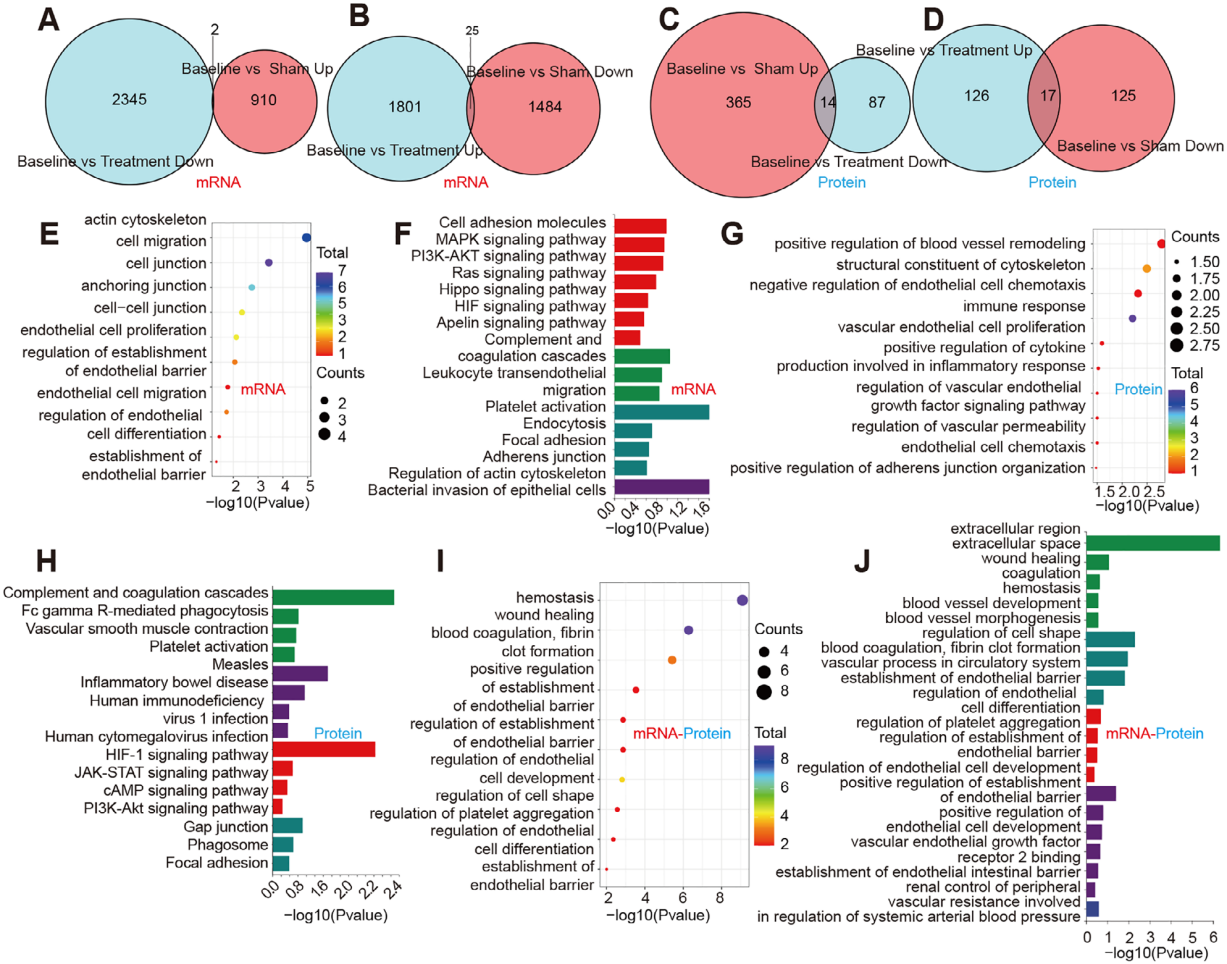
Concordant transcript‐protein changes and pathway enrichment associated with Galectin‐3 adsorption. (A)–(B) Venn diagrams showing differentially expressed transcripts exhibiting reversal with treatment relative to sham. Re‐regulated transcripts were defined as genes that changed in opposite directions in baseline vs. sham and baseline vs. treatment comparisons: (A) downregulated in baseline vs. treatment and upregulated in baseline vs. sham; (B) upregulated in baseline vs. treatment and downregulated in baseline vs. sham. (C)–(D) Venn diagrams showing differentially abundant proteins meeting the same reversal criteria: (C) downregulated in baseline vs. treatment and upregulated in baseline vs. sham; (D) upregulated in baseline vs. treatment and downregulated in baseline vs. sham. (E)–(F) GO enrichment analysis of re‐regulated transcripts (E) and re‐regulated proteins (F). Dot size indicates the number of mapped features per term (Counts), and color indicates enrichment significance (–log10 *P*). (G)–(H) KEGG pathway enrichment analysis of re‐regulated transcripts (G) and re‐regulated proteins (H); bar length indicates enrichment significance (–log10 *P*), and bar colors denote KEGG BRITE Level 1 functional categories: teal, Cellular Processes; purple, Human Diseases; red, Environmental Information Processing; dark blue, Metabolism; green, Organismal Systems. (I)–(J) GO (I) and KEGG (J) enrichment analyses of molecules exhibiting concordant directionality between the transcriptome and proteome (mRNA‐Protein).

Directional overlap analysis showed that only a small fraction of genes and proteins changed in opposite directions between the sham‐associated and treatment‐associated signatures (mRNA overlaps: 2 and 25; protein overlaps: 14 and 17) (Figure 5A–D). These findings indicate that Gal‐3 apheresis does not simply “reverse” the sham‐associated molecular changes; rather, it is associated with a more selective pattern of re‐regulation that preferentially involves pathways governing endothelial adhesion/junction organization and endothelial migration/permeability control (Figure 5E–H). In addition, integrative transcriptome‐proteome analysis identified 53 concordantly regulated molecules enriched in leukocyte transendothelial migration, cell adhesion molecules, and complement/coagulation pathways (Figure 5I–J, Figure S5), further highlighting Gal‐3 removal in the regulation of neutrophil endothelial interactions and preservation of vascular barrier function in sepsis.

### 2.8 Gal‐3 Apheresis Attenuates Chemokine Induction, Neutrophil Activation, and Multi‐Organ Neutrophil Infiltration

**FIGURE 6:**
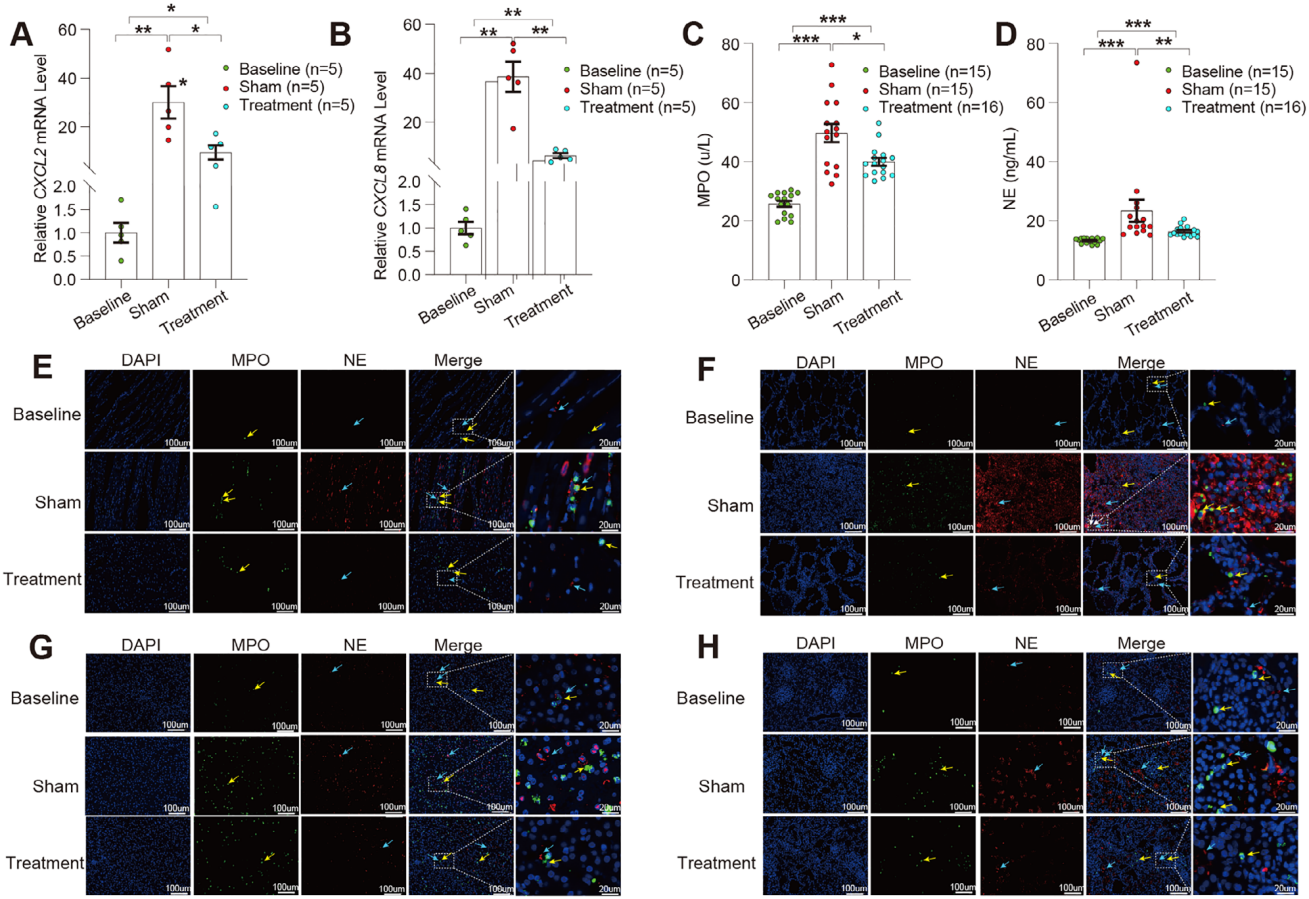
Neutrophil‐associated transcriptional, circulating, and tissue‐level readouts following Galectin‐3 adsorption. (A)–(B) qRT‐PCR quantification of *CXCL2* (A) and *CXCL8* (B) mRNA expression in peripheral blood leukocytes from baseline, sham, and treatment groups (*n* = 5 per group). Gene expression was normalized to GAPDH and calculated using the 2^−ΔΔCt^ method. (C)–(D) Serum concentrations of myeloperoxidase (MPO) (C) and neutrophil elastase (NE) (D) measured in baseline, sham, and treatment groups (*n* as indicated). (E)–(H) Representative immunofluorescence images of heart (E), lung (F), liver (G), and kidney (H) showing MPO (green) and NE (red) staining across baseline, sham, and treatment groups; nuclei were counterstained with DAPI (blue). Yellow arrows indicate MPO‐positive signal, and blue arrows indicate NE‐positive signal. Scale bars: 100 µm (main images) and 20 µm (magnified insets). Data are presented as individual values with bars indicating mean ± SEM. Prespecified pairwise comparisons were performed using two‐tailed Mann–Whitney *U* tests. *p < 0.05, **p < 0.01, ***p < 0.001.

Multi‐omics pathway enrichment consistently highlighted neutrophil chemotaxis and degranulation as dominant LPS‐responsive programs modulated by Gal‐3 removal. Accordingly, transcriptional profiling demonstrated that Gal‐3 apheresis markedly attenuated the early chemotactic response. While sham animals exhibited robust C–X–C motif chemokine ligand 2 (*CXCL2*) and C–X–C motif chemokine ligand 8 (*CXCL8*) upregulation post‐LPS (both *p* < 0.01 vs. baseline), Gal‐3 apheresis substantially suppressed both transcripts relative to sham (both *p* < 0.01; Figure 6A–B), consistent with reduced leukocyte recruitment signaling. Concordantly, circulating markers of neutrophil effector activation were significantly reduced in treated animals at 3 h, with lower myeloperoxidase (MPO) and neutrophil elastase (NE) (*p* < 0.001 and *p* < 0.01, respectively; Figure 6C–D), supporting attenuation of degranulation and extracellular trap release. Tissue‐level validation across heart, lung, liver, and kidney further demonstrated a coherent reduction in neutrophil accumulation, with decreased MPO and NE immunoreactivity throughout examined organs (Figure 6E–H). Together, these data indicate that Gal‐3 apheresis suppresses the chemokine‐driven recruitment axis and downstream neutrophil activation, translating into broadly reduced inflammatory infiltration across key target organs.

### 2.9 Gal‐3 Apheresis Reduces Endothelial Activation and Preserves Barrier Function

**FIGURE 7:**
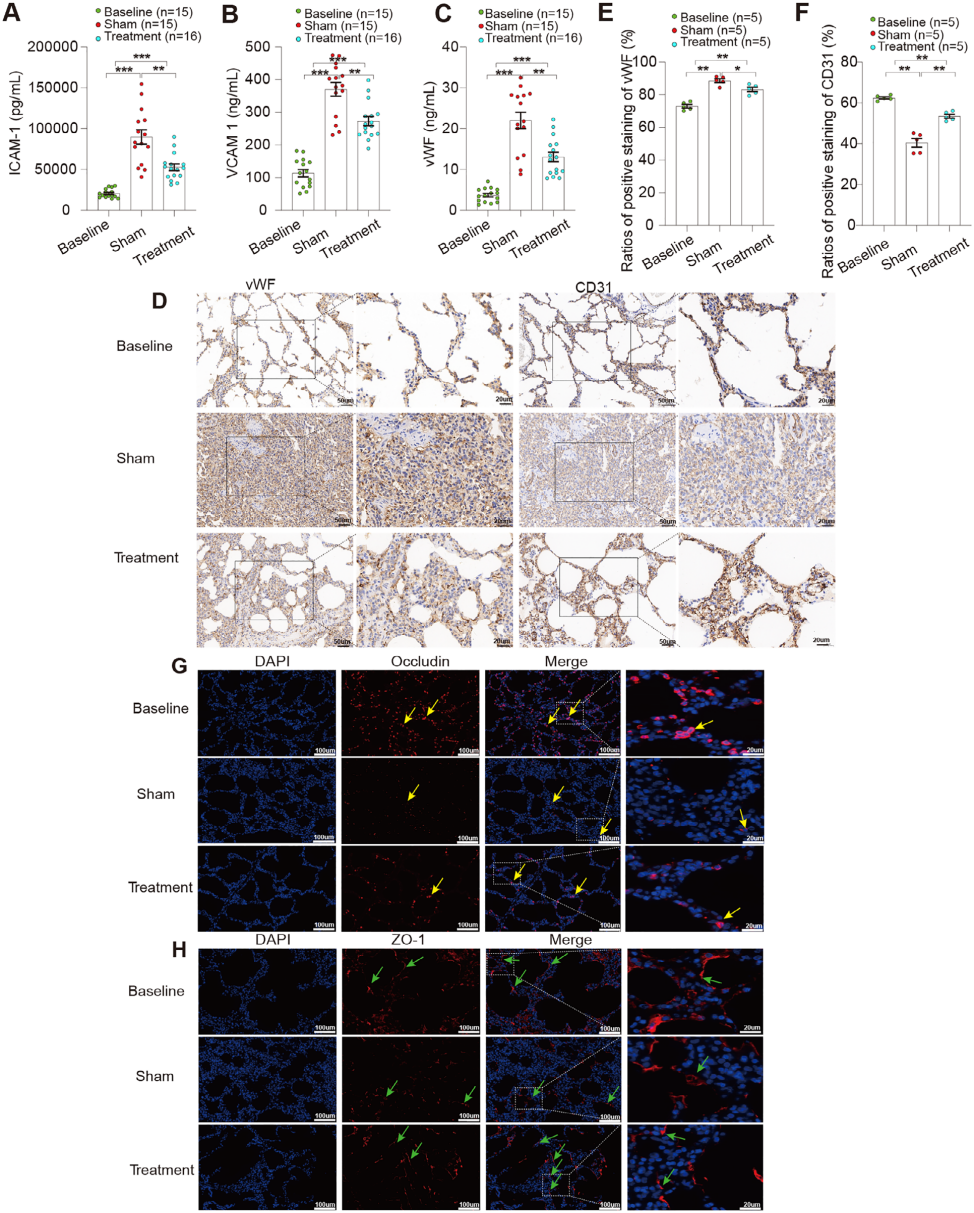
Endothelial injury markers and pulmonary vascular barrier readouts associated with Galectin‐3 adsorption. (A)–(C) Serum concentrations of endothelial‐associated markers ICAM‐1 (A), VCAM‐1 (B), and vWF (C) in baseline, sham, and treatment groups (*n* as indicated). (D) Representative IHC images of lung sections stained for vWF and CD31 across baseline, sham, and treatment groups, with corresponding higher‐magnification insets. (E)–(F) Quantification of the proportion of vWF‐positive (E) and CD31‐positive (F) staining in lung sections (*n* = 5 per group). (G)–(H) Representative immunofluorescence images of lung sections stained for tight‐junction proteins occludin (G) and ZO‐1 (H); nuclei were counterstained with DAPI. Yellow arrows indicate occludin‐positive signal, and green arrows indicate ZO‐1‐positive signal. Primary antibodies used for lung IHC were vWF (Proteintech, 27186‐1‐AP; 1:400) and CD31 (Abcam, ab182981; 1:2000), followed by secondary antibody (Abcam, ab205718; 1:2000). Scale bars: 50 µm (main images) and 20 µm (magnified insets). Data are presented as individual values with bars indicating mean ± SEM. Prespecified pairwise comparisons were performed using two‐tailed Mann–Whitney *U* tests. *p < 0.05, **p < 0.01, ***p < 0.001.

Given the close coupling between neutrophil effector activity and endothelial injury in endotoxemia, we assessed vascular activation and barrier integrity. In sham animals, circulating endothelial injury markers von Willebrand factor (vWF), vascular cell adhesion molecule‐1 (VCAM‐1), and intercellular adhesion molecule‐1 (ICAM‐1) rose sharply at 3 h post‐LPS (all *p* < 0.001 vs. baseline), indicating rapid endothelial activation and damage. Gal‐3 apheresis significantly blunted this response (all *p* < 0.01 vs. sham; Figure 7A–C), demonstrating preservation of the vascular interface. In the lung, immunohistochemical profiling corroborated these systemic findings: treated animals displayed lower vWF immunoreactivity (*p* < 0.05 vs. sham) alongside preserved expression of the endothelial marker cluster of differentiation 31 (CD31) (*p* < 0.01; Figure 7D–F), consistent with maintained endothelial integrity. Importantly, treatment was also associated with higher levels of the tight‐junction proteins occludin and zonula occludens‐1 (ZO‐1) (Figure 7G–H), supporting improved junctional stability and barrier preservation. Collectively, Gal‐3 removal mitigated endothelial activation while sustaining structural components of intercellular junctions, a pattern consistent with reduced vascular leak susceptibility during systemic inflammation.

### 2.10 Gal‐3 Apheresis Dampens PI3K‐AKT and Hypoxia Signaling

**FIGURE 8:**
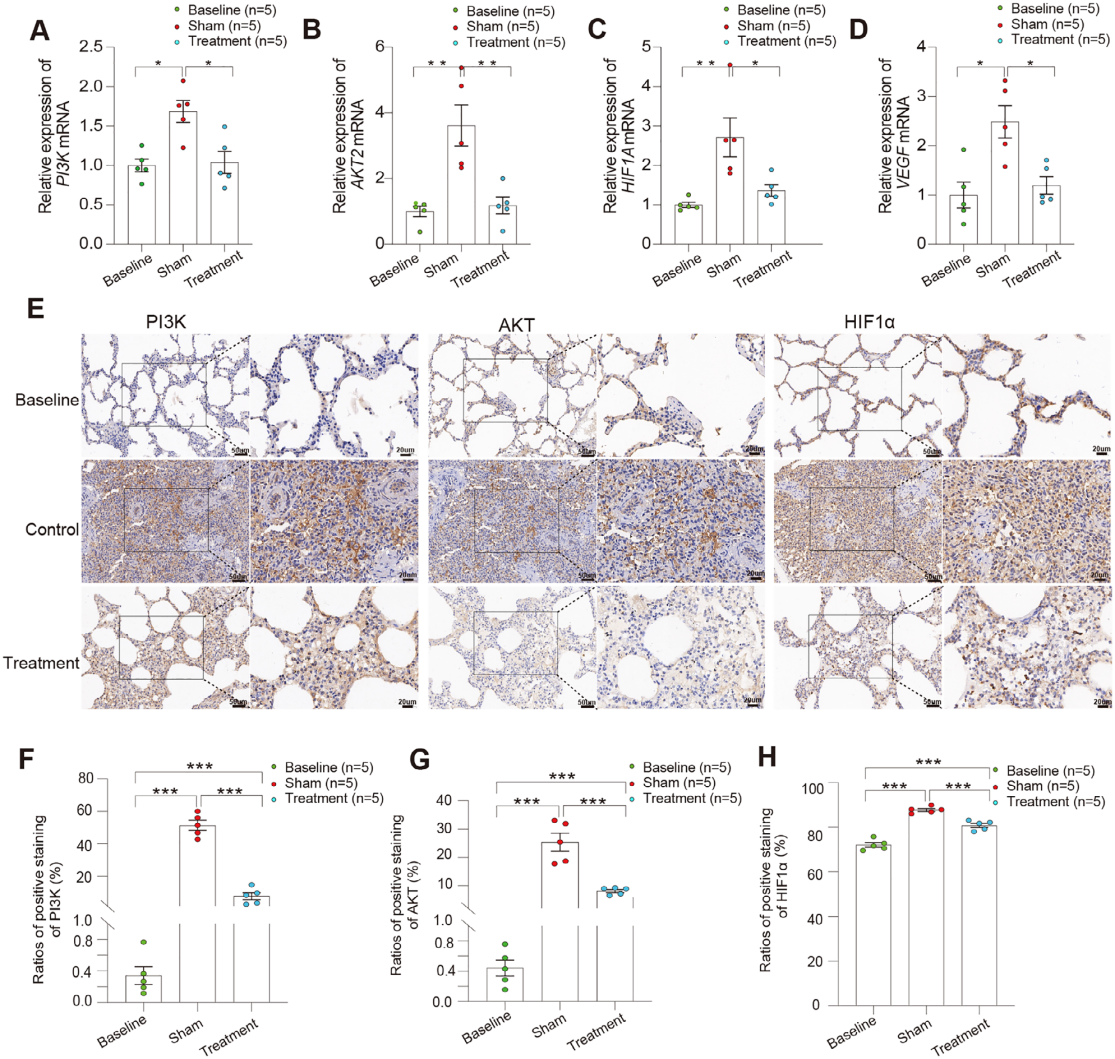
PI3K‐AKT and HIF‐1 pathway‐related readouts in representative tissue following Galectin‐3 adsorption. (A)–(D) qRT‐PCR quantification of *PI3K* (A), *AKT2* (B), *HIF1A* (C), and *VEGFA* (D) mRNA expression measured 3 h after LPS challenge in baseline, sham, and treatment groups (*n* = 5 per group). Gene expression was normalized to GAPDH and calculated using the 2^−ΔΔCt^ method, expressed as fold change relative to baseline. (E) Representative IHC images from lung tissue (shown as a representative organ) stained for PI3K, AKT, and HIF‐1α across baseline, sham, and treatment groups, with corresponding higher‐magnification insets. (F)–(H) Quantification of the proportion of PI3K‐positive (F), AKT‐positive (G), and HIF‐1α‐positive (H) staining in lung sections (*n* = 5 per group). Scale bars: 50 µm (main images) and 20 µm (magnified insets). Primary antibodies for IHC were PI3K (Proteintech, 60225‐1‐Ig; 1:400), AKT (Proteintech, 66444‐1‐Ig; 1:400), and HIF‐1α (Bioss, Bs‐0737R; 1:500). Secondary antibodies were goat anti‐mouse IgG (Abcam, ab6789; 1:1000) for PI3K and AKT, and goat anti‐rabbit IgG (Abcam, ab205718; 1:2000) for HIF‐1α. Data are presented as individual values with bars indicating mean ± SEM. Prespecified pairwise comparisons were performed using two‐tailed Mann–Whitney *U* tests.*p < 0.05, **p < 0.01, ***p < 0.001.

To connect these phenotypes to upstream regulatory programs suggested by omics enrichment, we evaluated transcript and protein levels of PI3K‐AKT and hypoxia‐related pathways. Sham animals showed increased expression of *PI3K* and *AKT2* transcripts following LPS challenge, whereas Gal‐3 apheresis significantly reduced both (*PI3K p* < 0.05; *AKT2 p* < 0.01 vs. sham; Figure 8A–B), indicating suppression of a central inflammatory‐survival signaling axis. In parallel, key hypoxia‐inducible factor 1‐alpha (*HIF‐1A*) and vascular endothelial growth factor A (*VEGFA*) transcripts were elevated in sham animals but were significantly attenuated with treatment (both *p* < 0.05; Figure 8C–D), consistent with reduced hypoxic stress signaling. Protein‐level validation by IHC aligned with transcriptomic changes, demonstrating lower PI3K, AKT, and HIF‐1α staining in the treatment group relative to controls (Figure 8E–H). These convergent findings support a model in which Gal‐3 removal limits PI3K‐AKT activation and downstream hypoxia‐adaptation, potentially contributing to reduced hypoxic stress, improved endothelial stability, and reduced inflammatory tissue injury.

## 3 Discussion

This study examines the potential therapeutic relevance of Gal‐3 removal in sepsis by integrating clinical observations with experimental validation. In a prospective clinical cohort, downtrending Gal‐3 levels were associated with improved 28‐day survival, suggesting that Gal‐3 dynamics are associated with improved clinical outcomes. Guided by this observation, we evaluated Gal‐3 apheresis in two preclinical sepsis models, each demonstrating higher survival and reduced organ injury in treated animals compared with sham controls. The CLP rat and endotoxemic porcine models were used to capture complementary dimensions of human sepsis: infection‐driven host responses and survival biology, as well as severe cardiopulmonary and endothelial dysfunction secondary to a hyperinflammatory response. The concordant findings across both models, together with longitudinal observations in patients, strengthen translational relevance while addressing model‐specific limitations. Additionally, while the observational human cohort data cannot infer causality, the consistency across both human and preclinical results highlights Gal‐3 as a potential therapeutic target.

Sepsis has proven to be impervious to existing therapeutic interventions, and extracorporeal blood‐purification approaches have shown inconsistent results [18, 24]. Recent meta‐analyses evaluating broad‐spectrum blood filtration revealed modest pooled risk ratios for mortality reduction [19, 25]. Similarly, general adsorption strategies carry only low‐certainty evidence of mortality benefit, likely reflecting the inherent limitations of broad‐spectrum removal that indiscriminately eliminates both beneficial and pathogenic mediators [20, 26]. In contrast, removal of an upstream mediator may offer a more focused means of modulating dysregulated host responses [14, 27].

The mechanistic patterns observed in our study are consistent with this hypothesis. Gal‐3 is an upstream damage‐associated molecular pattern (DAMP) implicated in multiple features of sepsis pathobiology, including leukocyte activation and endothelial dysfunction. We found that Gal‐3 removal attenuated key features of neutrophil‐endothelial dysfunction, a hallmark of sepsis‐related organ injury [28, 29]. Treated animals exhibited reduced NET formation, decreased neutrophil infiltration, and lower chemokine concentrations. These findings are consistent with known Gal‐3‐dependent activation pathways [14, 30]. Importantly, antibacterial defense appeared preserved, supporting the concept that Gal‐3 removal may recalibrate, rather than suppress, innate immunity [27, 31]. Although neutrophils represented the most prominent cellular signal in our analyses, Gal‐3 biology extends beyond a single immune subset. Transcriptomic profiling revealed changes in gene modules associated with macrophage activation and leukocyte adhesion, as well as alterations in selected T‐cell activation‐related signatures. These patterns align with previously described roles of Gal‐3 in shaping monocyte trafficking [32, 33], macrophage phenotype [33, 34], and T‐cell survival [35, 36]. The findings suggest the possibility of broader immunoregulatory effects on both the innate and adaptive immune system, and warrant further mechanistic studies with functional assays to delineate cell‐type‐specific contributions.

Gal‐3 removal also influenced endothelial structure and microvascular function, representing a critical dimension of the therapeutic effect beyond immune modulation [37, 38]. Treated animals demonstrated lower levels of endothelial injury markers and preservation of tight junction proteins (ZO‐1, claudin‐5, occludin), indicating maintenance of endothelial barrier integrity [39]. These structural findings paralleled physiological improvements, including reduced extravascular lung water, decreased pulmonary edema, improved lactate clearance, and lower vasopressor requirements—indicators of preserved microvascular perfusion and tissue oxygen delivery [40, 41].

In our clinical cohort, the temporal trajectory of Gal‐3 distinguished survivors from non‐survivors, in contrast to the trajectory of IL‐6, which downtrended similarly between groups. The divergent temporal patterns of Gal‐3 and IL‐6 among the patient cohort, in combination with our pre‐clinical findings, suggest that Gal‐3 may reflect an upstream process in the immune dysregulation of sepsis, which is more closely linked to organ dysfunction. Gal‐3 has been implicated in processes such as leukocyte activation, endothelial injury, and microvascular dysfunction, and its sustained elevation in non‐survivors may represent continued activation of these upstream pathways or a failure to re‐establish homeostasis.

Beyond immune and endothelial effects, Gal‐3 removal modulated metabolic pathways relevant to sepsis pathophysiology [42, 43]. Transcriptomic and proteomic analyses revealed normalization of PI3K‐AKT and HIF‐1α signaling, suggesting improved metabolic adaptation and cellular oxygen utilization [44, 45]. These pathway changes are biologically plausible given that Gal‐3 has been implicated in metabolic dysregulation during critical illness, including mitochondrial dysfunction and altered glucose metabolism [46]. Additionally, our findings raise the possibility that Gal‐3 removal may reduce ferroptosis‐related injury, although definitive validation will require dedicated mechanistic studies [47].

Importantly, the multidimensional effects demonstrated in this study suggest that the observed survival benefit stems from a synergistic mechanism in which Gal‐3 removal simultaneously addresses multiple pathophysiological domains: recalibration of immune responses; restoration of endothelial barrier function and microvascular perfusion; and normalization of metabolic pathways critical for cellular energetics and stress adaptation. These findings indicate that the observed survival differences are associated with a constellation of biologic changes, including immune modulation, endothelial barrier preservation, and metabolic pathway shifts. The convergence of these effects represents an intervention that directly addresses the underlying pathophysiology of sepsis, using a column that seamlessly integrates into an existing plasmapheresis platform and can be used independently or in combination with other blood filtration treatments.

Several study limitations warrant acknowledgment. The clinical study was observational and cannot establish causality between dynamic changes of Gal‐3 and outcomes. Nevertheless, the consistent pattern across both human observation and interventional animal models, in which Gal‐3 removal improved survival, supports the therapeutic rationale. Secondly, the 24‐h observation period in pigs, though sufficient for demonstrating acute benefit, cannot address longer‐term outcomes. Finally, the LPS‐induced sepsis paradigm may not fully capture the complexity of human polymicrobial infections with active bacterial replication [48, 49]; however, it remains a commonly used model in large animals [50, 51, 52, 53, 54]. Notably, our findings were also demonstrated in the widely‐used CLP model of polymicrobial sepsis. While our intervention successfully controlled excessive inflammatory responses in this preclinical model, clinical use will include co‐administration of antimicrobials to address the inciting pathogen [38, 39].

In conclusion, through convergence of clinical observation and preclinical interventions, our study demonstrated the therapeutic potential of Gal‐3 apheresis in the targeted treatment of sepsis. The improvement in survival, organ dysfunction, and tissue damage, related to coordinated restoration of immune regulation and endothelial barrier function, provides a robust translational foundation for future clinical trials.

## 4 Materials and Methods

### 4.1 Clinical Observation

This prospective observational study was approved by Wuhan University Zhongnan Hospital (NO.2019141) and used to observe the relationship between the dynamic changes of Gal‐3 and the outcome in septic patients. All septic patients diagnosed with Sepsis‐3 criterion [55] in ICU from August 2020 to December 2022 were enrolled. Exclusion criteria included immunodeficiency, pregnancy, active renal replacement therapy, organ transplantation, age <18 years, and expected survival <24 h. Primary outcome: 28‐day all‐cause mortality. Blood samples were collected on Days 1–3 for measurement of serum Gal‐3 and IL‐6 by enzyme‐linked immunosorbent assay (ELISA; R&D Systems, Minneapolis, MN, USA).

### 4.2 Gal‐3 Apheresis System Development

The XGAL‐3 system (Eliaz Therapeutics Inc., Santa Rosa, CA, USA) employed high‐affinity anti‐Gal‐3 monoclonal antibodies (K_D = 0.361 nM) conjugated to activated agarose matrices. Chimeric immunoglobulin G1 (IgG1) antibody in Chinese hamster ovary K1 (CHO‐K1) cells achieved >99% purity with endotoxin <0.04 EU/mg. For rat applications, species‐specific anti‐rat Gal‐3 antibodies were developed using identical production systems. Validation studies in human plasma demonstrated >95% Gal‐3 removal efficiency without capacity saturation at clinically relevant plasma‐to‐column ratios (60:1 and 240:1) (Table S8, Figures S6–S7) [22]. The XGAL‐3 column seamlessly integrates into the widely‐used Terumo Spectra Optia plasmapheresis platform. The system utilizes venous access and can operate at lower blood flow rates than blood filtration treatments, allowing it to be used independently or in combination with other blood filtration treatments.

### 4.3 Gal‐3 Apheresis Study in Septic Rats

The protocol (NO.2018024, Wuhan University) followed Animal Research: Reporting of In Vivo Experiments (ARRIVE) guidelines. Male Sprague‐Dawley rats (*n* = 48, 400–600 g) underwent 7‐day acclimation and randomization to Gal‐3 apheresis (*n* = 28) or sham apheresis (*n* = 20). Polymicrobial sepsis was induced via CLP under isoflurane anesthesia: 25% cecal ligation with 4‐0 silk suture, two 20‐gauge needle punctures, and closure with fluid resuscitation (20 mL/kg saline) plus analgesia (buprenorphine 0.05 mg/kg).

At 60 min post‐CLP, whole blood apheresis (0.8–1.0 mL/min via femoral‐jugular circuit, 120 min) processed approximately∼1.6–2.0 blood volumes through anti‐rat Gal‐3 antibody columns (treatment) or sham columns (sham). Temperature was maintained at 37°C ± 0.5°C, and anticoagulation was used with heparin (100 U/kg bolus, 50 U/kg/h). Seven‐day survival was monitored with standardized humane endpoints. Blood samples were collected at baseline, post‐apheresis, and 24‐h intervals.

### 4.4 Gal‐3 Apheresis in Porcine Sepsis Model

#### 4.4.1 Experimental Design and Animal Model

This randomized, controlled, investigator‐blinded study used 31 male Bama miniature pigs (28–32 kg) per institutional protocols (NO. ZN2022053) and ARRIVE guidelines. Animals were randomized to Gal‐3 apheresis (*n* = 16) or sham apheresis (*n* = 15) using sealed envelopes. The sample size provided 80% power to detect 40% survival difference (*α* = 0.05).

### 4.5 Instrumentation and Sepsis Induction

Sepsis induction employed escalating LPS protocols: initial phase (2–4 µg/kg/h), recovery period (30 min), and acceleration phase (progressive escalation to 24 µg/kg/h), consistently achieving severe sepsis criteria, defined as > 30% MAP reduction, tachycardia, systemic inflammation [22] (Figure S8). Gal‐3 apheresis was initiated during the acceleration phase using the XGAL‐3 apheresis column with the Terumo BCT Spectra Optia platform (Terumo BCT, Lakewood, CO, USA) with 25 mL/min plasma flow for a 3.5 h period. Sham animals underwent identical extracorporeal procedures, including vascular access, circuit priming, and plasma circulation through inactive sham adsorption columns (identical matrices without Gal‐3 antibodies). All clinical personnel performing hemodynamic assessments and data collection remained blinded to treatment allocation throughout the study period.

#### 4.5.1 Comprehensive Monitoring and Supportive Care

Hemodynamics were measured with the Pulse Index Continuous Cardiac Output (PiCCO) monitoring system (thermodilution calibration per manufacturer and repeated after major physiologic changes), including cardiac index(CI), stroke volume index (SVI), stroke volume/pulse pressure variation (SVV/PPV), and volumetric preload indices, namely global end‐diastolic volume index (GEDI), and intrathoracic blood volume index (ITBVI). ELWI and metabolic parameters (including arterial blood gas, lactate, and electrolytes) were recorded hourly. All animals received standardized supportive care, including volume‐controlled mechanical ventilation (tidal volume 6–8 mL/kg, positive end‐expiratory pressure 5 cm H_2_O) under deep sedation with neuromuscular blockade during hemodynamic assessments. Fluid management followed evidence‐based protocols applied uniformly across both groups, with administration contingent upon demonstrated fluid responsiveness [56, 57, 58]. When fluid responsiveness was confirmed, balanced crystalloid (5 mL/kg) was administered over 5–10 min with subsequent reassessment. Fluid therapy was considered successful when SVI increased by ≥10%–15% and/or SVV/PPV decreased to <10%–12% without ELWI elevation >1 mL/kg or oxygenation deterioration. Vasopressor support with norepinephrine was titrated to maintain MAP ≥ 70% of individual baseline values. MAP served as the primary vasopressor endpoint and a trigger for fluid responsiveness testing rather than mandating fluid administration. Treatment allocation codes were revealed only after completion of all data collection and preliminary statistical analysis to ensure investigator blinding integrity.

#### 4.5.2 Multi‐Omics Analysis and Biomarker Assessment

All mechanistic experiments were performed using an LPS‐induced porcine sepsis model. Peripheral blood samples were collected at Baseline, 3 h after LPS infusion (Control), and 3 h after LPS infusion following Gal‐3 apheresis (Treatment). Peripheral blood leukocytes were used for transcriptomic and RT‐qPCR analyses, and serum was used for proteomic, metabolomic, and biomarker measurements. RNA sequencing was employed using Illumina NovaSeq 6000 platform, and differential gene expression was analyzed with DESeq2 (false discovery rate (FDR) <0.05, fold change >1.5). Proteomic analysis used data‐independent acquisition mass spectrometry with MaxQuant processing. Metabolomic profiling employed ultra‐performance liquid chromatography–mass spectrometry (UPLC‐MS) with pathway enrichment analysis using Kyoto Encyclopedia of Genes and Genomes (KEGG) databases.

### 4.6 Serum Biomarker Quantification

Serum biomarkers were quantified via validated enzyme‐linked immunosorbent assays (ELISAs): Gal‐3 (FinTest), IL‐6 (R&D Systems), myeloperoxidase (Jiancheng), neutrophil elastase (FinTest), ICAM‐1 (FinTest), VCAM‐1, and vWF(All in Shanghai Enzyme‐linked Biotechnology Co., Ltd.).

#### 4.6.1 Gene Expression Analysis (RT‐qPCR)

We extracted leukocyte RNA using TRIzol reagent (Invitrogen, Carlsbad, CA, USA) and reverse transcribed using ReverTra Ace qPCR RT Kit (TOYOBO, Osaka, Japan). Reverse transcription quantitative polymerase chain reaction (RT‐qPCR) was performed with UltraSYBR Mixture (CWBIO, Beijing, China). Gene expression was normalized to GAPDH and calculated using the 2^−ΔΔC^ *^t^* method. Primer sequences are provided in Table S9.

#### 4.6.2 Histopathological Analysis

At 24 h, surviving pigs were euthanized, and heart, liver, lung, and kidney tissues were harvested, formalin‐fixed, paraffin‐embedded, sectioned (4 µm), and H&E‐stained. A pathologist blinded to group allocation assigned semiquantitative injury scores using prespecified, organ‐specific criteria: lung scores were calculated as the sum of five domains (0–4 each; total 0–20), and heart/liver/kidney scores as the sum of four domains (0–4 each; total 0–16). For each animal, five randomly selected high‐power fields (HPFs) per organ from standardized sampling sites were scored and averaged to yield one value per organ per animal.

Immunostaining on paraffin sections was performed using DAB‐based immunohistochemistry (IHC) or immunofluorescence (IF), as indicated. DAB‐IHC was performed for vWF, CD31, PI3K, AKT, and HIF‐1α, and signals were detected using an HRP‐DAB system with hematoxylin counterstaining; no‐primary and/or isotype controls were run in parallel. IF was performed for MPO, neutrophil elastase (NE), occludin, and ZO‐1, with nuclei counterstained using DAPI (5 µg/mL, 10 min at room temperature in the dark; PBS, pH 7.4, washes 3×5 min). Image acquisition and ImageJ‐based quantification were conducted under blinded conditions. Complete reagent information, including sources/catalog numbers and working dilutions for all primary/secondary antibodies, is provided in Table S10.

#### 4.6.3 Statistical Analysis

All analyses were performed using R software (version 4.3.2). Normality was assessed using the Shapiro–Wilk test. Normally distributed continuous variables are presented as mean ± SEM with 95% confidence intervals and compared using Student's t‐tests (or paired *t*‐tests for within‐subject comparisons). Non‐normally distributed data were expressed as median (interquartile range) with 95% confidence intervals for medians calculated using bootstrap resampling (10,000 iterations), and analyzed using Mann–Whitney U test (or Wilcoxon signed‐rank test for paired comparisons). Effect sizes were calculated to quantify the magnitude of differences: Cliff's Delta (δ) for sepsis versus control comparisons and rank‐biserial correlation (r) for survivor versus non‐survivor comparisons, with 95% confidence intervals estimated using bootstrap methods (5000 iterations). Categorical variables were presented as frequencies (percentages) and compared using Fisher's exact test or chi‐square test. Survival curves were generated using the Kaplan‐Meier method and compared using the log‐rank test. All tests were two‐sided, and *p* < 0.05 was considered statistically significant.

## Author Contributions

Z.S. designed and conducted experiments, analyzed data, and wrote the manuscript. J.Q. performed animal experiments and conducted specific assays. S.P. executed animal protocols and collected data. Y.H. assisted with animal experiments and data collection. A.E. contributed to study design and data interpretation. G.C. critically reviewed the manuscript. I.E. and Z.P. conceived and supervised the study, interpreted results, and revised the manuscript. All authors approved the final version.

## Funding

The study was partially supported by the National Natural Science Foundation of China (NO. 82241039).

## Ethics Statement

The prospective observational clinical study was approved by the Ethics Committee of Zhongnan Hospital of Wuhan University (Approval No. 2019141). All animal experiments were approved by the Animal Care and Use Committee of Wuhan University (Approval Nos. 2018024 and ZN2022053) and conducted in accordance with the ARRIVE guidelines.

## Conflicts of Interest

I.E. is the inventor of Gal‐3 extracorporeal depletion and the developer of the XGAL‐3 apheresis column. G.C., Z.P., and A.E. are scientific advisors to Eliaz Therapeutics Inc. The other authors declared no conflict of interest.

## Supporting information




**Supporting File 1**: mco270659‐sup‐0001‐SuppMat.docx

## Data Availability

The RNA‐seq data generated in this study have been deposited in the CNCB‐NGDC (CNGBdb) repository under accession CNP0008631 (DOI: 10.26036/CNP0008631). The proteomics data have been deposited in CNCB‐NGDC (CNGBdb) under accession CNP0008629 (DOI: 10.26036/CNP0008629). The metabolomics data have been deposited in EBI MetaboLights under accession MTBLS13537. Data can be accessed by contacting the corresponding author upon reasonable request and completion of appropriate institutional data‐sharing agreements.
