# Correction to “Therapeutic Galectin‐3 Apheresis Improves Sepsis Outcomes through Coordinated Neutrophil Modulation and Endothelial Barrier Preservation: A Translational Study”

**DOI:** 10.1002/mco2.70853

**Published:** 2026-08-05

Z. Sun, J. Qu, S. Peng, et al. “Therapeutic Galectin‐3 Apheresis Improves Sepsis Outcomes Through Coordinated Neutrophil Modulation and Endothelial Barrier Preservation: A Translational Study.” *MedComm* 7, no. 4 (2026): e70659.

In the title page, the co‐first author statement was missing. This should have read: “Both Zhongyi Sun and Jiachen Qu shared the first authorship.”

We apologize for this error.

