# Supplementary material for: Therapeutic Galectin‐3 Apheresis Improves Sepsis Outcomes Through Coordinated Neutrophil Modulation and Endothelial Barrier Preservation: A Translational Study
Source: MedComm (2020). 2026 Mar 15;7(4):e70659. doi: 10.1002/mco2.70659 (PMC13042957; doi:10.1002/mco2.70659)
Supplement: Supplementary file 1 — Supporting File 1: mco270659‐sup‐0001‐SuppMat.docx [file MCO2-7-e70659-s001.docx]

**Therapeutic Galectin-3 apheresis improves sepsis outcomes through coordinated neutrophil modulation and endothelial barrier preservation: a translational study**Zhongyi Sun^1, 2^, Jiachen Qu^1, 2^, Sheng Peng^1, 2^, Yanan Hu^1, 2^, Amity Eliaz^3^, Glenn M Chertow^4^, Isaac Eliaz^5*^ Zhiyong Peng^1, 2*^

1. Department of Critical Care Medicine, Zhongnan Hospital of Wuhan University, Wuhan, Hubei, China.

2. Clinical Research Center of Hubei Critical Care Medicine, Wuhan, Hubei, China.

3. Department of Neurology, University of California, San Francisco, California, USA.

4. Department of Medicine, Epidemiology and Population Health, and Health Policy, Stanford University School of Medicine, Stanford, California, USA.

5. Amitabha Medical Clinic and Healing Center, Santa Rosa, California, USA.

*Isaac Eliaz & Zhiyong Peng share senior authorship.

Correspondence:

Dr Isaac Eliaz, MD, MS and Dr Zhiyong Peng, MD, PhD, FCCM

Isaac Eliaz, Amitabha Medical Clinic and Healing Center, 398 Tesconi Ct, Santa Rosa, CA 95401, USA (isaac.eliaz@gmail.com).

Zhiyong Peng, Department of Critical Care Medicine, Zhongnan Hospital of Wuhan University, Wuhan, Hubei Province, 430071, China; Clinical Research Center of Hubei Critical Care Medicine, Wuhan, Hubei, China (pengzy5@hotmail.com).

**Table S1**. Baseline Characteristics and Laboratory Findings of Healthy Volunteers and Patients with Sepsis

| Variables | Healthy Volunteers （n=27） | Septic Patients  （n=87） | *P* |
| --- | --- | --- | --- |
| Age （years） | 54（50-60） | 64（53-74） | 0.004 |
| Female sex, n （%） | 10（37.03） | 36（41.4） | 0.88 |
| Gal-3 Day1 （ng/ml） | 9.7（8.4-10.4） | 20.7（14.6-29.2） | < 0.001 |
| Gal-3 Day2 （ng/ml） |  | 15.5（12.17-19.6） | < 0.001 |
| Gal-3 Day3 （ng/ml） |  | 15.1（10.9-19.9） | < 0.001 |
| IL-6 Day1 （pg/ml） | 1.2（0.7-1.5） | 130.9（39.8-337.8） | < 0.001 |
| IL-6 Day2 （pg/ml） |  | 66.0（30.9-173.1） | < 0.001 |
| IL-6 Day3 （pg/ml） |  | 29.9（14.3-66.4） | < 0.001 |

**Notes:** Continuous variables are presented as median (IQR); categorical variables as number (%).
P values were calculated using the Mann–Whitney U test for continuous data and the χ² test or Fisher’s exact test for categorical data.

**Table S2**. Baseline Characteristics and Laboratory Parameters of Septic Patients Stratified by Survival Status

| Variable | Sepsis patients were grouped by survival | | |
| --- | --- | --- | --- |
|  | Survivors（n=55） | Non-Survivors （n=32） | *P* |
| Age （years） | 64（54-72） | 69（57-76） | 0.16 |
| Female sex, n（%） | 23（41.82） | 13（40.63） | 1 |
| Gal-3 Day1（ng/ml） | 21.82（16.9-33.2） | 17.5（10.6-25.2） | 0.03 |
| Gal-3 Day2（ng/ml） | 16.2（11.8-18.6） | 14.0（12.6-22.7） | 0.52 |
| Gal-3 Day3（ng/ml） | 12.4（9.4-17.6） | 18.1（14.6-25.1） | 0.007 |
| IL6 Day1（pg/ml） | 149.6（54.9-407.4） | 111.8（39.7-221.5） | 0.52 |
| IL6 Day2（pg/ml） | 59.4（26.0-127.5） | 75.3（44.8-200.8） | 0.25 |
| IL6 Day3（pg/ml） | 27.1（13.0-64.3） | 37.6（23.0-157.6） | 0.37 |
| LAC（mmol/L） | 2.2（1.5-3.4） | 2.8（1.8-4.4） | 0.164 |
| APACHE II score | 23（14-28） | 26（16.5-32） | 0.18 |
| SOFA score | 8（5.5-13） | 8（5-13） | 0.85 |

**Notes:** Continuous variables are expressed as median (IQR); categorical variables as number (%).

P values were calculated using the Mann–Whitney U test for continuous variables and the χ² test or Fisher’s exact test for categorical variables.

Abbreviations: LAC, lactate; SOFA, Sequential Organ Failure Assessment; APACHE II, Acute Physiology and Chronic Health Evaluation II.

**Table S3.** Condition comparison of the endotoxemic pig

| Group | Variable | Baseline | Post LPS | Post LPS 1h | Post LPS 2h | Post LPS 3h |
| --- | --- | --- | --- | --- | --- | --- |
| Sham | MAP | 85（80-87） | 50（48-51） | 70（69-71） | 69（68-71） | 69（68-71） |
|  | HR | 63（62-78） | 98（78-110） | 96（92-114） | 101（94-108） | 99（87-128） |
| Treatment | MAP | 84（81-86） | 50（49-51） | 70（69-71） | 71（67-73） | 71（68-73） |
|  | HR | 65（63-86） | 87（71-101） | 105（88-111） | 97（94-112） | 95（85-109） |

HR: Heart Rate; MAP: Mean Arterial Pressure

**Table S4**. Comparison of blood biochemical values

| Group | Variable | Baseline | Post LPS | Post LPS 1h | Post LPS 2h | Post LPS 3h |
| --- | --- | --- | --- | --- | --- | --- |
| Sham | Crea | 77.8±21.9 | 79.1±36.8 | 114.2±124.2 | 88.8±35.4 | 92.4±23.9 |
|  | AST | 129.2±217.7 | 65.0±126.9 | 61.3±132.4 | 65.0±112.9 | 69.7±132.9 |
|  | ALT | 42.5±25.2 | 34.3±21.0 | 34.4±16.2 | 30.5±16.6 | 34.7±19.7 |
| Treatment | Crea | 95.7±21.5 | 90.4±24.3 | 97.4±29.2 | 102.1±26.0 | 98.8±24.1 |
|  | AST | 30.6±38.3 | 21.7±14.8 | 28.3±19.0 | 36.3±43.2 | 32.9±20.1 |
|  | ALT | 37.7±11.2 | 28.5±7.0 | 28.8±7.0 | 27.2±6.2 | 27.2±6.9 |

Crea: Creatinine; AST: Aspartate Aminotransferase; ALT: Alanine Aminotransferase

**Table S5.** Comparison of pulmonary ventilation function parameters

| Group | Variable | Baseline | Post LPS | Post LPS 1h | Post LPS 2h | Post LPS 3h |
| --- | --- | --- | --- | --- | --- | --- |
| Sham | Pplat | 10.67±2.60 | 14.11±3.89 | 15.78±4.18 | 16.33±4.69 | 17.56±5.29 |
|  | PIP | 20.00±6.61 | 19.78±6.26 | 23.11±6.43 | 24.44±7.35 | 23.11±7.74 |
|  | Pmean | 5.89±5.51 | 7.44±7.88 | 8.22±8.29 | 11.11±7.57 | 10.44±8.11 |
|  | R | 14.11±4.37 | 14.11±3.02 | 16.11±4.54 | 16.44±4.36 | 17.56±5.48 |
|  | CL | 30.67±5.24 | 25.22±9.60 | 22.53±13.96 | 23.03±13.20 | 22.64±12.16 |
| Treatment | Pplat | 13.15±4.06 | 13.54±4.01 | 15.00±4.02 | 15.08±3.97 | 14.62±3.62 |
|  | PIP | 21.77±5.20 | 23.46±7.59 | 23.92±6.25 | 22.77±8.44 | 24.38±6.50 |
|  | Pmean | 6.31±3.22 | 8.54±10.12 | 8.77±10.01 | 8.92±9.47 | 8.62±8.69 |
|  | R | 12.38±2.18 | 15.69±6.01 | 16.85±5.97 | 16.46±6.57 | 16.31±5.86 |
|  | CL | 29.38±7.74 | 24.34±11.02 | 21.99±9.04 | 22.94±9.58 | 21.65±7.43 |

Pplat: Plateau Pressure; PIP: Peak Inspiratory Pressure; Pmean: Mean Airway Pressure; R: Airway Resistance; CL: Lung Compliance.

**Table S6.** Comparison of General Clinical Parameters Between Sham and Treatment Groups in the Endotoxemic Pig Model at Different Time Points.

| **Parameter** | **Group** | **Baseline** | **Post LPS** | **Post LPS 1h** | **Post LPS 2h** | **Post LPS 3h** |
| --- | --- | --- | --- | --- | --- | --- |
| Mean arterial pressure (mmHg) | Sham | 70.9 ± 16.4 | 67.1 ± 20.0 | 67.7 ± 13.1 | 59.7 ± 3.9 | 62.3 ± 11.8 |
|  | Treatment | 68.2 ± 8.0 | 60.1 ± 10.0 | 63.5 ± 9.3 | 62.9 ± 10.1 | 67.1 ± 14.8 |
| Heart rate (beats/min) | Sham | 67.0 ± 8.0 | 97.0 ± 19.0 | 101.0 ± 14.0 | 102.0 ± 11.0 | 103.0 ± 14.0 |
|  | Treatment | 75.0 ± 17.0 | 84.0 ± 25.0 | 104.0 ± 20.0 | 107.0 ± 23.0 | 97.0 ± 20.0 |
| Central venous pressure (mmHg) | Sham | 12.3 ± 5.5 | 14.1 ± 4.8 | 13.7 ± 3.1 | 13.4 ± 3.7 | 15.5 ± 3.3 |
|  | Treatment | 10.2 ± 5.5 | 9.8 ± 4.9 | 10.3 ± 5.0 | 10.6 ± 5.0 | 11.7 ± 5.1 |
| Cardiac index (L/min/m²) | Sham | 2.4 ± 0.9 | 3.2 ± 1.1 | 3.2 ± 1.1 | 3.1 ± 1.3 | 3.0 ± 1.3 |
|  | Treatment | 2.6 ± 0.7 | 2.6 ± 0.5 | 2.35 ± 0.4 | 2.6 ± 0.7 | 2.6 ± 0.8 |
| Systemic vascular resistance index (dyn·s·cm⁻⁵·m²) | Sham | 1874.9 ± 732.1 | 1484.3 ± 410.8 | 1374.4 ± 358.6 | 1288.3 ± 384.3 | 1476.0 ± 701.3 |
|  | Treatment | 2021.1 ± 571.0 | 1884.6 ± 477.2 | 1848.6 ± 506.3 | 1629.6 ± 400.8 | 1745.8 ± 499.1 |
| Global ejection fraction (%) | Sham | 36.3 ± 7.0 | 34.8 ± 5.2 | 34.5 ± 6.2 | 33.7 ± 7.8 | 31.1 ± 8.2 |
|  | Treatment | 35.8 ± 8.7 | 34.9 ± 7.6 | 33.1 ± 8.2 | 33.4 ± 6.5 | 33.3 ± 8.8 |
| Cardiac power index (W/m²) | Sham | 0.3 ± 0.2 | 0.5 ± 0.3 | 0.5 ± 0.2 | 0.4 ± 0.2 | 0.4 ± 0.2 |
|  | Treatment | 0.4 ± 0.1 | 0.3 ± 0.2 | 0.3 ± 0.1 | 0.3 ± 0.1 | 0.4 ± 0.2 |
| Stroke volume variation (%) | Sham | 9.2 ± 2.7 | 10.0 ± 2.7 | 10.7 ± 4.4 | 9.6 ± 2.6 | 10.0 ± 4.4 |
|  | Treatment | 11.9 ± 7.3 | 10.9 ± 4.0 | 13.4 ± 4.9 | 13.8 ± 6.0 | 12.2 ± 6.3 |
| Extravascular lung water (mL/kg) | Sham | 13.4 ± 3.2 | 18.0 ± 5.2 | 20.8 ± 5.3 | 22.4 ± 7.7 | 24.6 ± 6.4 |
|  | Treatment | 14.6 ± 4.7 | 14.4 ± 3.7 | 14.3 ± 2.2 | 15.9 ± 3.7 | 16.5 ± 3.4 |
| Pulmonary vascular permeability index | Sham | 3.1 ± 0.5 | 3.4 ± 1.05 | 4.3 ± 1.4 | 4.1 ± 1.2 | 4.6 ± 1.7 |
|  | Treatment | 3.1 ± 0.7 | 3.5 ± 0.9 | 3.9 ± 0.9 | 3.9 ± 0.9 | 3.8 ± 1.2 |
| Oxygen saturation, SpO₂ (%) | Sham | 95.9 ± 3.4 | 96.2 ± 4.6 | 95.8 ± 5.0 | 93.1 ± 12.4 | 92.5 ± 10.3 |
|  | Treatment | 98.0 ± 1.7 | 98.9 ± 0.9 | 94.4 ± 15.5 | 94.9 ± 15.0 | 96.0 ± 10.3 |

* Values are mean ± SD. LPS denotes lipopolysaccharide.

**Table S7.** Comparison of blood routine values

| Variable | Sham | | | | | Treatment | | | | |
| --- | --- | --- | --- | --- | --- | --- | --- | --- | --- | --- |
|  | Base | Post LPS | Post LPS 1h | Post LPS 2h | Post LPS 3h | Base | Post LPS | Post LPS 1h | Post LPS 2h | Post LPS 3h |
| WBC（10^^9^） | 18.0±6.7 | 4.3±3.3 | 3.2±1.7 | 3.7±2.0 | 4.6±2.6 | 18.9±6.5 | 5.4±2.7 | 4.8±2.1 | 4.2±2.8 | 4.6±2.4 |
| RBC（10^^12^） | 5.8±0.8 | 6.3±1.0 | 6.3±1.0 | 8.5±6.1 | 6.5±1.0 | 6.7±1.5 | 6.8±0.7 | 7.0±1.0 | 6.6±1.2 | 6.8±1.1 |
| HGB（G/L） | 90.2±12.0 | 104.2±14.9 | 94.4±17.2 | 98.2±12.9 | 98.5±16.9 | 109.5±31.2 | 109.4±18.0 | 112.9±23.4 | 104.1±26.1 | 109.9±24.6 |
| HCT（%） | 29.9±3.7 | 34.9±5.3 | 32.1±5.8 | 33.4±4.5 | 33.5±6.1 | 35.7±9.8 | 36.2±5.2 | 37.5±6.9 | 35.0±7.7 | 36.7±7.5 |
| MCV（FL） | 51.5±2.4 | 51.9±2.7 | 51.6±2.6 | 51.7±2.9 | 51.7±2.7 | 52.9±4.0 | 52.7±3.8 | 53.0±3.9 | 53.1±3.9 | 53.5±4.2 |
| MCH（PG） | 15.5±1.0 | 15.5±1.1 | 17.8±7.9 | 15.2±1.1 | 15.2±1.0 | 16.1±1.4 | 15.9±1.5 | 15.9±1.6 | 15.6±1.9 | 16.0±1.7 |
| MCHC（G/L） | 301.3±6.8 | 298.9±11.3 | 283.9±11.1 | 294.4±12.6 | 294.6±12.6 | 306.0±7.4 | 302.0±11.0 | 299.5±10.0 | 294.8±4.6 | 298.3±9.8 |
| PLT（10^^9^） | 507.7±83.0 | 316.3±112.4 | 293.7±141.4 | 276.3±104.4 | 255.1±81.2 | 421.4±160.9 | 261.8±105.7 | 242.9±121.7 | 236.2±94.5 | 233.9±82.9 |
| MPV（FL） | 7.4±1.6 | 7.3±1.6 | 13.3±17.9 | 7.5±1.5 | 13.7±19.1 | 7.5±1.5 | 7.4±1.5 | 7.3±1.4 | 7.3±1.1 | 7.4±1.3 |

WBC: White Blood Cell count; RBC: Red Blood Cell count; HGB: Hemoglobin; HCT: Hematocrit; MCV: Mean Corpuscular Volume; MCH: Mean Corpuscular Hemoglobin; MCHC: Mean Corpuscular Hemoglobin Concentration; PLT: Platelet count; MPV: Mean Platelet Volume

To assess transcriptomic relationships among the Baseline, Sham, and Treatment groups, we performed principal component analysis (PCA) on the RNA-seq expression matrix. The PCA revealed clear separation of the three conditions, with tight within-group clustering of biological replicates, indicating high intragroup concordance and minimal technical variability (Fig. S1A). The first two principal components explained the majority of variance (PC1: 63.4%; PC2: 19.2%), highlighting robust, condition-associated transcriptional differences.

Differential expression was quantified using DESeq2. Genes were defined as differentially expressed using the pre-specified criteria |log_2_(fold change)∣>1 and P < 0.05. Across pairwise comparisons, we identified 2,421 differentially expressed genes (DEGs) in Baseline vs Sham, 3,886 DEGs in Sham vs Treatment, and 1,486 DEGs in Baseline vs Treatment (Fig. S1B). Overlap analysis indicated both shared and comparison-specific signatures: 269 DEGs were common to all three contrasts, while substantial pairwise overlaps were observed between Baseline vs Sham and Sham vs Treatment (1,543 DEGs), Sham vs Treatment and Baseline vs Treatment (803 DEGs), and Baseline vs Sham and Baseline vs Treatment (181 DEGs) (Fig. S1B). Volcano plots illustrate the magnitude and statistical significance of DEGs for each comparison, with upregulated and downregulated genes highlighted (Fig. S1C–E).

| 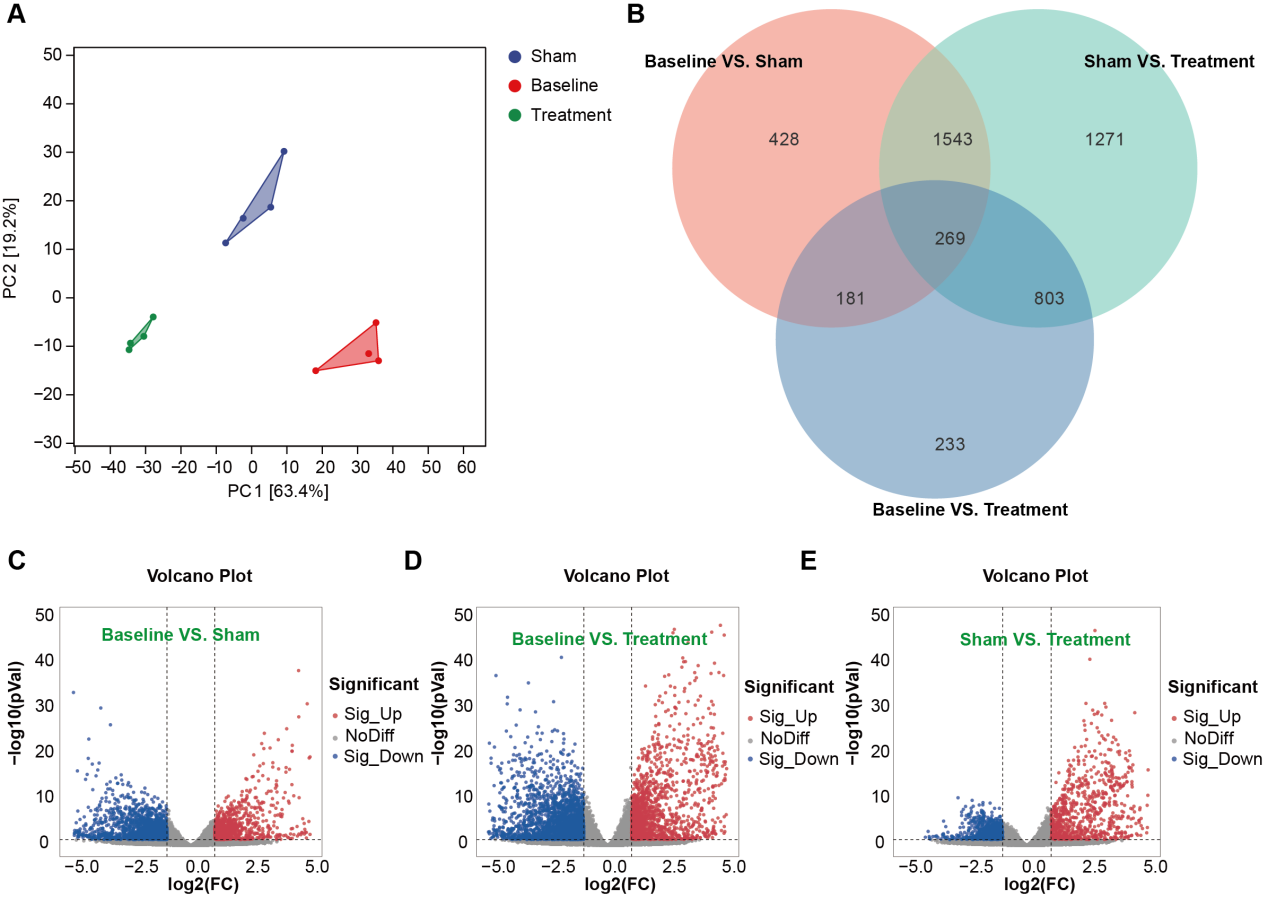 |
| --- |
| **Figure S1.** Transcriptomic separation and differential gene expression across Baseline, Sham, and Treatment conditions. (A) PCA of transcriptome-wide expression profiles. Samples cluster by condition, demonstrating high within-group similarity and clear separation among Baseline, Sham, and Treatment groups. Axes indicate the proportion of variance explained (PC1, 63.4%; PC2, 19.2%). Shaded polygons represent the convex hull encompassing samples within each group. (B) Venn diagram summarizing the overlap of DEGs across the three pairwise comparisons (Baseline vs Sham, Sham vs Treatment, Baseline vs Treatment). Numbers denote DEGs unique to each contrast and shared between/among contrasts. (C–E) Volcano plots for (C) Baseline vs Sham, (D) Baseline vs Treatment, and (E) Sham vs Treatment. Genes meeting the DEG criteria (\|log_2_FC∣>1 and P<0.05; DESeq2) are colored as significantly upregulated (red) or downregulated (blue); non-significant genes are shown in grey. |

Unsupervised PCA of the quantitative proteome showed clear condition-dependent separation, with tight clustering of replicates within each group (PC1: 33.24%; PC2: 13.15%), indicating high proteomic reproducibility and robust differences among Baseline, Sham, and Treatment (Fig. S2A). Protein coverage was broadly shared across groups: 1,186 proteins were quantified in all three conditions, while a smaller fraction was group-specific (Sham-only: 110; Baseline-only: 56; Treatment-only: 46). Pairwise overlaps were 96 (Sham vs. Baseline), 109 (Sham vs. Treatment), and 80 (Baseline vs. Treatment), corresponding to 1,501 proteins in Sham, 1,418 in Baseline, and 1,421 in Treatment (Fig. S2B). Differential abundance analyses (|log2FC| > 1, P < 0.05) identified proteins with significant increases or decreases across all pairwise comparisons, as summarized by volcano plots for Baseline vs Sham, Baseline vs Treatment, and Sham vs Treatment (Fig. S2C–E).

| 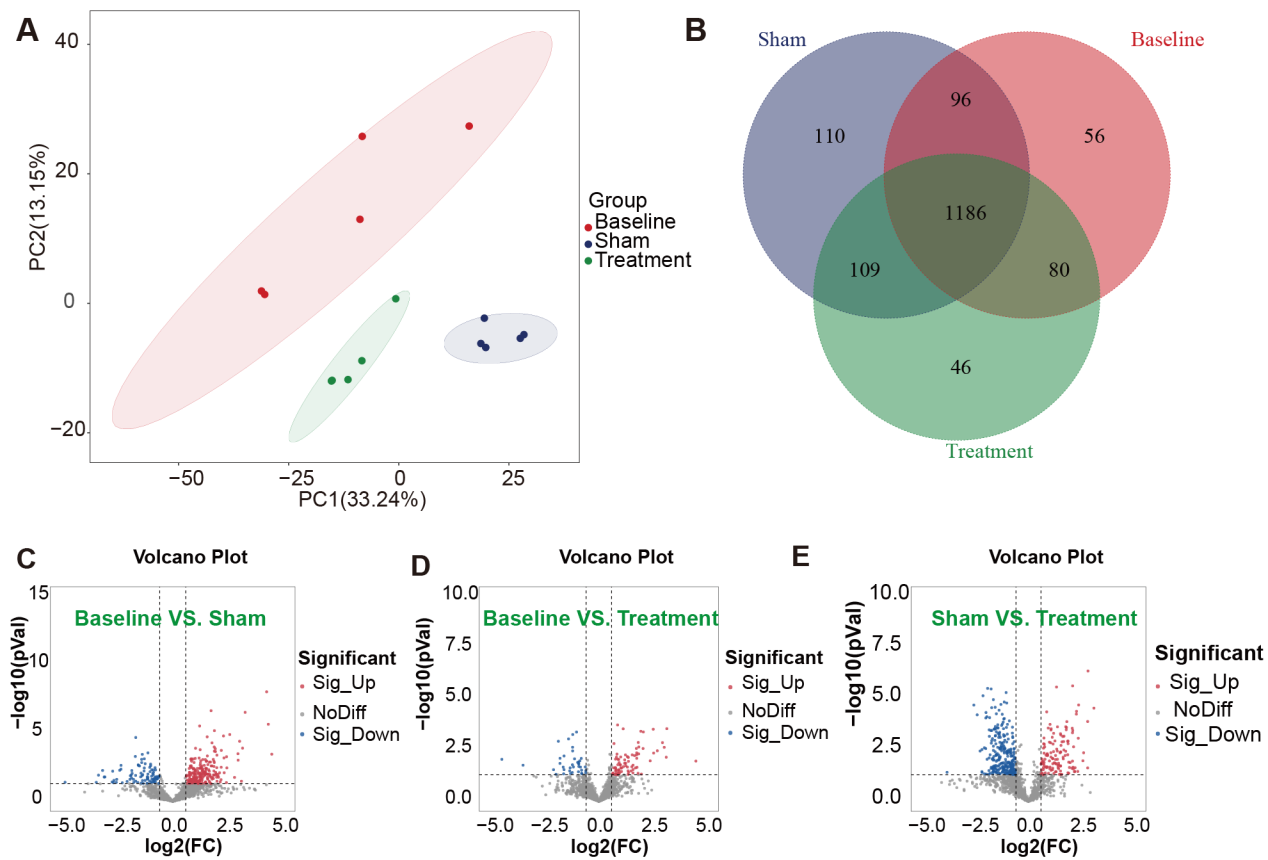 |
| --- |
| **Figure S2.** Proteome-wide separation, protein coverage overlap, and differential abundance across Baseline, Sham, and Treatment groups. (A) PCA of proteomic profiles across Baseline, Sham, and Treatment groups (PC1, 33.24%; PC2, 13.15%); ellipses indicate within-group dispersion. (B) Venn diagram showing overlap of quantified proteins across groups (shared: 1,186; Sham-only: 110; Baseline-only: 56; Treatment-only: 46; pairwise overlaps as indicated). (C-E) Volcano plots of differential protein abundance for (C) Baseline vs Sham, (D) Baseline vs Treatment, and (E) Sham vs Treatment. Dashed lines denote thresholds (\|log2FC\| = 1; P = 0.05). Upregulated and downregulated proteins are highlighted relative to non-significant proteins. |

PCA of LC–MS metabolomic profiles demonstrated robust, condition-associated separation across all pairwise contrasts in both electrospray ionization modes (Positive: POS, and Negative: NEG), with tight within-group clustering indicative of high analytical and biological consistency (Fig. S3A-F). In POS, Baseline segregated from Sham (PC1 21.4%, PC2 12.6%), Baseline separated from Treatment (PC1 31.3%, PC2 11.4%), and Sham separated from Treatment (PC1 20.6%, PC2 17.9%). In NEG, separation was similarly evident and explained by comparable or greater variance (Baseline vs Sham: PC1 26.1%, PC2 18.2%; Baseline vs Treatment: PC1 37.3%, PC2 22.8%; Sham vs Treatment: PC1 28.5%, PC2 22.4%). Collectively, these ordinations support a reproducible and systematic remodeling of the metabolome across experimental conditions, consistently captured in both ionization polarities.

| 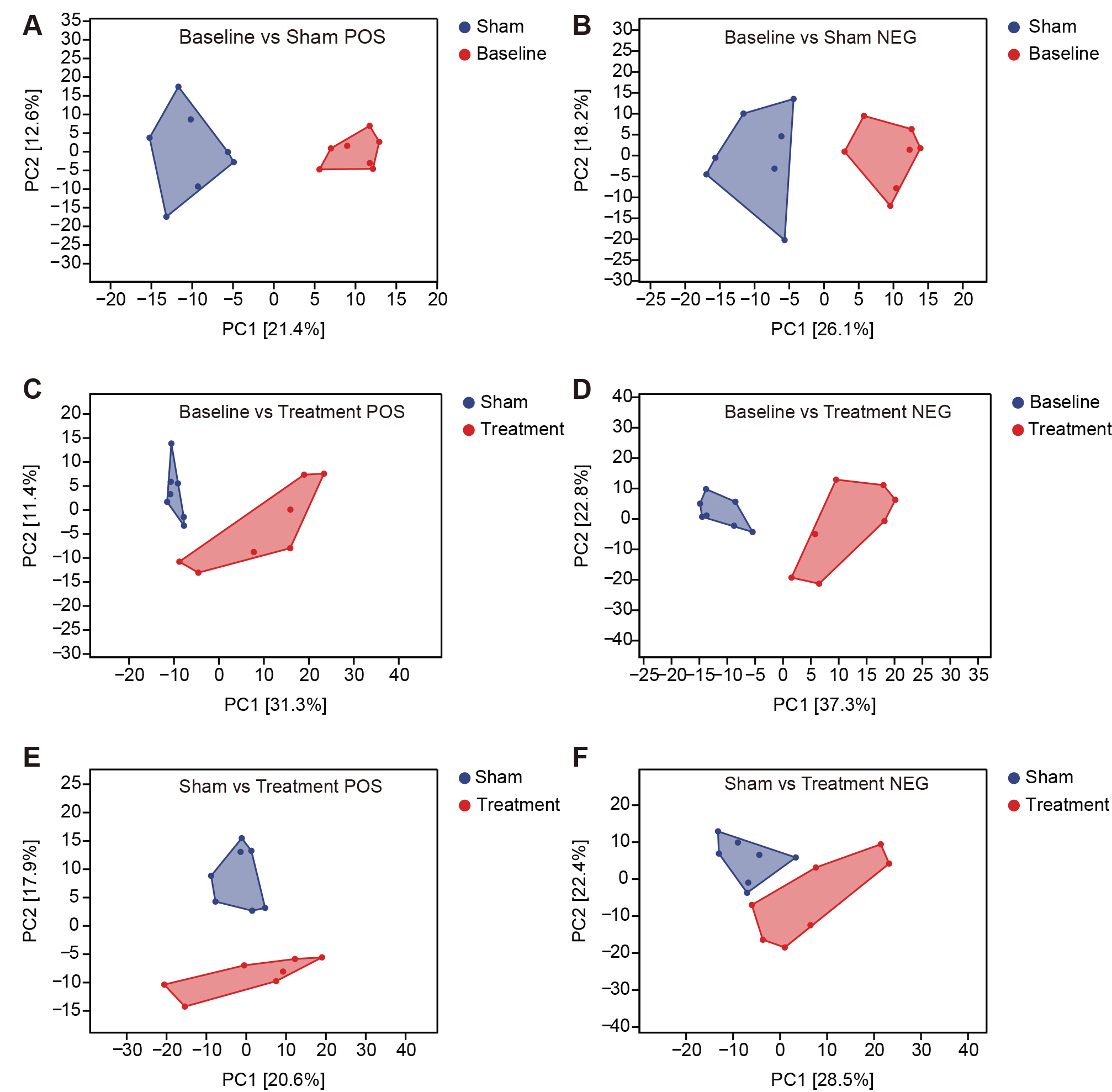 |
| --- |
| **Figure S3.** Pairwise PCA of metabolomic profiles in positive and negative ionization modes. (A,B) Baseline vs Sham in POS and NEG modes, (C,D) Baseline vs Treatment in POS and NEG modes, (E,F) Sham vs Treatment in POS and NEG modes. |

Differential metabolites were defined using a combined multivariate–univariate strategy, applying OPLS-DA variable importance in projection (VIP) > 1 together with two-sided P < 0.05. Using these prespecified criteria, volcano plots demonstrate robust metabolic perturbations across all pairwise contrasts (Baseline vs Sham, Baseline vs Treatment, and Sham vs Treatment) in both negative (NEG) and positive (POS) ionization modes, with significant features distributed on both sides of the fold-change axis (Figure S4A–F).

Overlap analysis indicates the presence of both conserved and contrast-specific metabolic signatures. In NEG mode, 142 (Baseline vs Sham), 212 (Baseline vs Treatment), and 133 (Sham vs Treatment) differential metabolites were identified, with 13 shared across all three contrasts (Figure S4G). In POS mode, totals were 130, 172, and 107, respectively, with 9 metabolites common to all contrasts (Figure S4H). When integrating both modes (ALL), 272, 384, and 240 differential metabolites were detected for the three comparisons, with 22 shared across all contrasts (Figure S4I). Collectively, these data support a core set of consistently dysregulated metabolites together with substantial condition-specific remodeling of the metabolome.

| 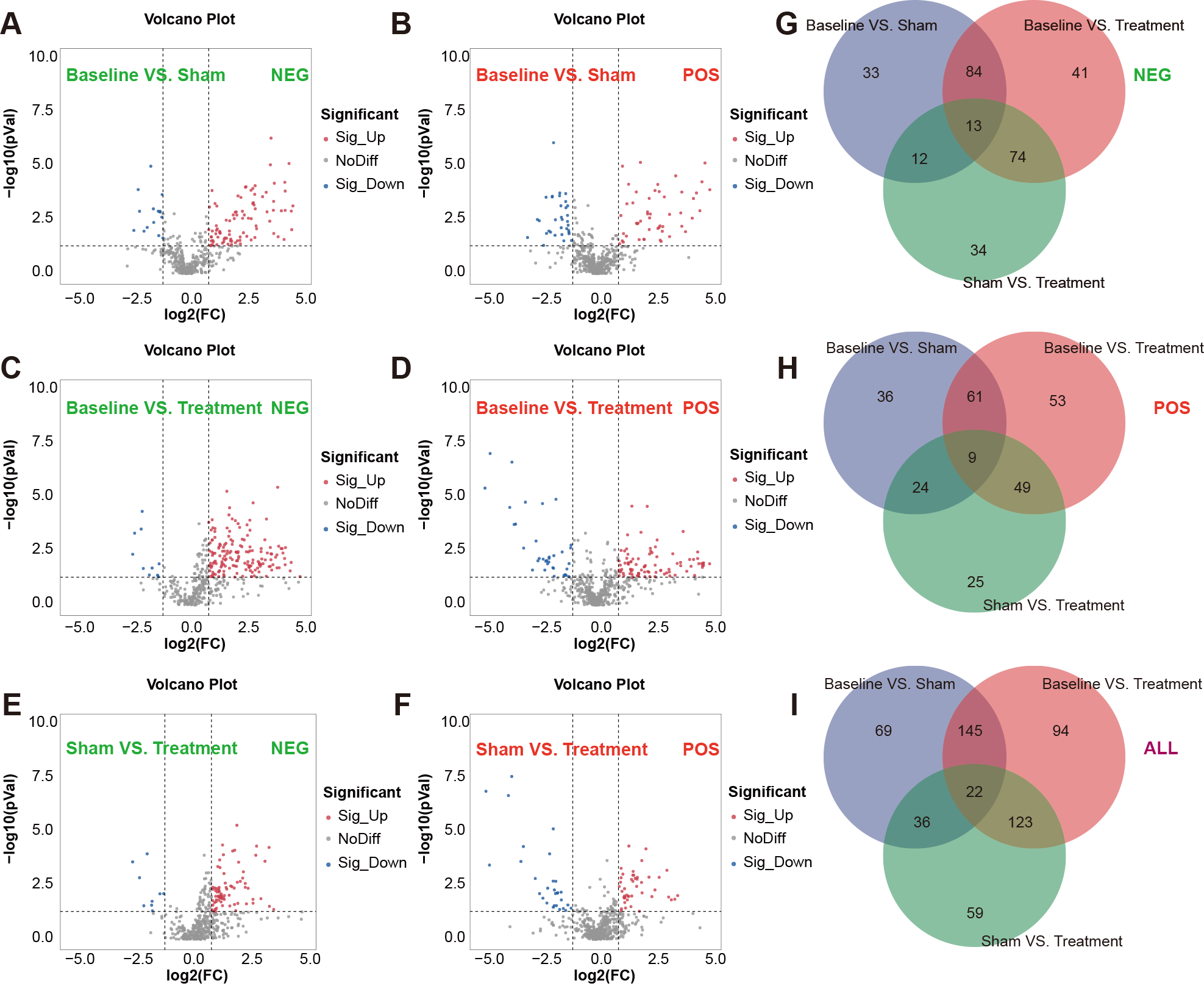 |
| --- |
| **Figure S4**. Differential metabolites identified and their overlap across contrasts. (A,C,E) Volcano plots for Baseline vs Sham, Baseline vs Treatment, and Sham vs Treatment in NEG mode. (B,D,F) Corresponding volcano plots in POS mode. Differential metabolites were defined as VIP > 1 (from OPLS-DA) and two-sided P < 0.05. Significantly increased and decreased metabolites are highlighted (red and blue, respectively); non-significant features are shown in grey. (G–I) Venn diagrams summarizing overlap of differential metabolites among the three pairwise contrasts in NEG (G), POS (H), and combined modes (ALL) (I). Numbers indicate metabolites unique to each contrast or shared between/among contrasts. |

To assess cross-platform concordance of the treatment-associated response, we integrated the Sham vs Treatment differential transcriptome and proteome. Among significant features, 4,107 transcripts and 430 proteins were identified as differentially regulated, and 53 molecules were shared between the two datasets based on unambiguous mRNA-protein mapping (Figure S5A). These overlapping features represent a high-confidence core set of treatment-responsive molecules detected at both the transcript and protein levels.

We next evaluated the relationship between transcript- and protein-level effect sizes by comparing log2 fold-changes for matched mRNA-protein pairs (Figure S5B). Overall, the global correlation was negligible (Pearson r = −0.029), indicating that treatment-associated changes in mRNA abundance do not uniformly translate into proportional protein changes across the full dataset, consistent with known post-transcriptional and post-translational regulation. Nevertheless, the subset of 53 concordantly differential mRNA-protein pairs highlights a biologically tractable, cross-validated signature that is most likely to reflect direct, robust regulation by Gal-3 apheresis.

| 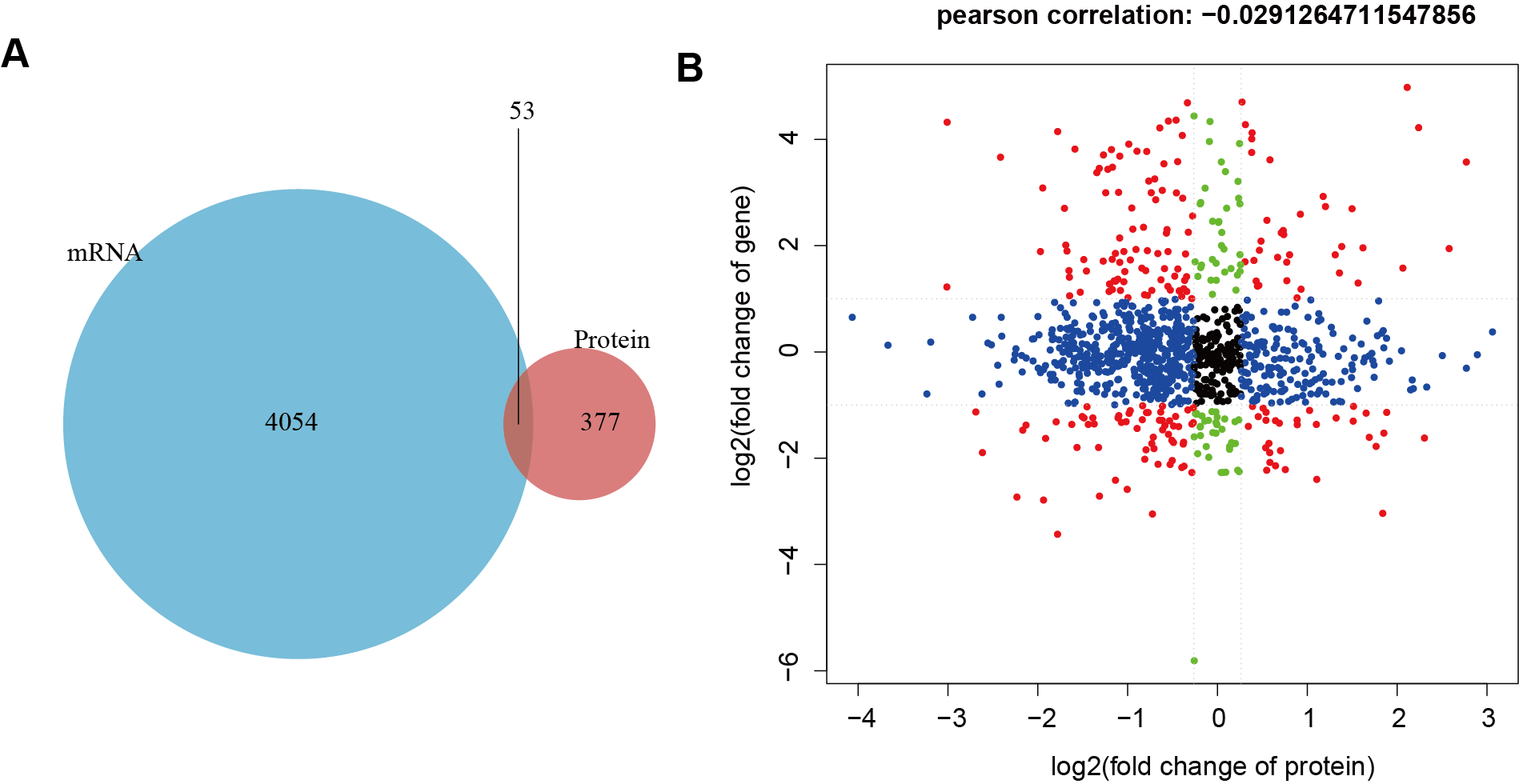 |
| --- |
| **Figure S5**. Integrated transcriptome–proteome analysis for Sham vs Treatment. (A) Venn diagram summarizing differential features that could be matched by gene/protein identifiers between datasets. Differential analysis identified 4,107 transcripts and 430 proteins in Sham vs Treatment, of which 53 mapped molecules were significant in both the transcriptome and proteome. (B) Scatter plot of matched mRNA-protein pairs showing the relationship between transcriptomic and proteomic effect sizes. The x-axis indicates log2(fold change) in protein abundance and the y-axis indicates log2(fold change) in mRNA expression (Sham vs Treatment). The overall Pearson correlation coefficient is shown at the top. Points are colored by significance category: red, significant in both transcriptome and proteome; blue, significant only at the protein level; green, significant only at the transcript level; black, not significant in either dataset. |

**Table S8.**

| **Molecule information** | |
| --- | --- |
| **Molecular type** | **mAb** |
| **Extinction coefficient** | **1.45** |
| **Molecular weight (kDa)** | **146** |
| **Expression** | |
| **Cell line** | **CHO-K1** |
| **Purification & QC** | |
| **Purification steps** | **MSS-POROS XS-UF/DF** |
| **Purity of SEC-HPLC (%)** | **100.00** |
| **Endotoxin (EU/mg)** | **< 0.037** |


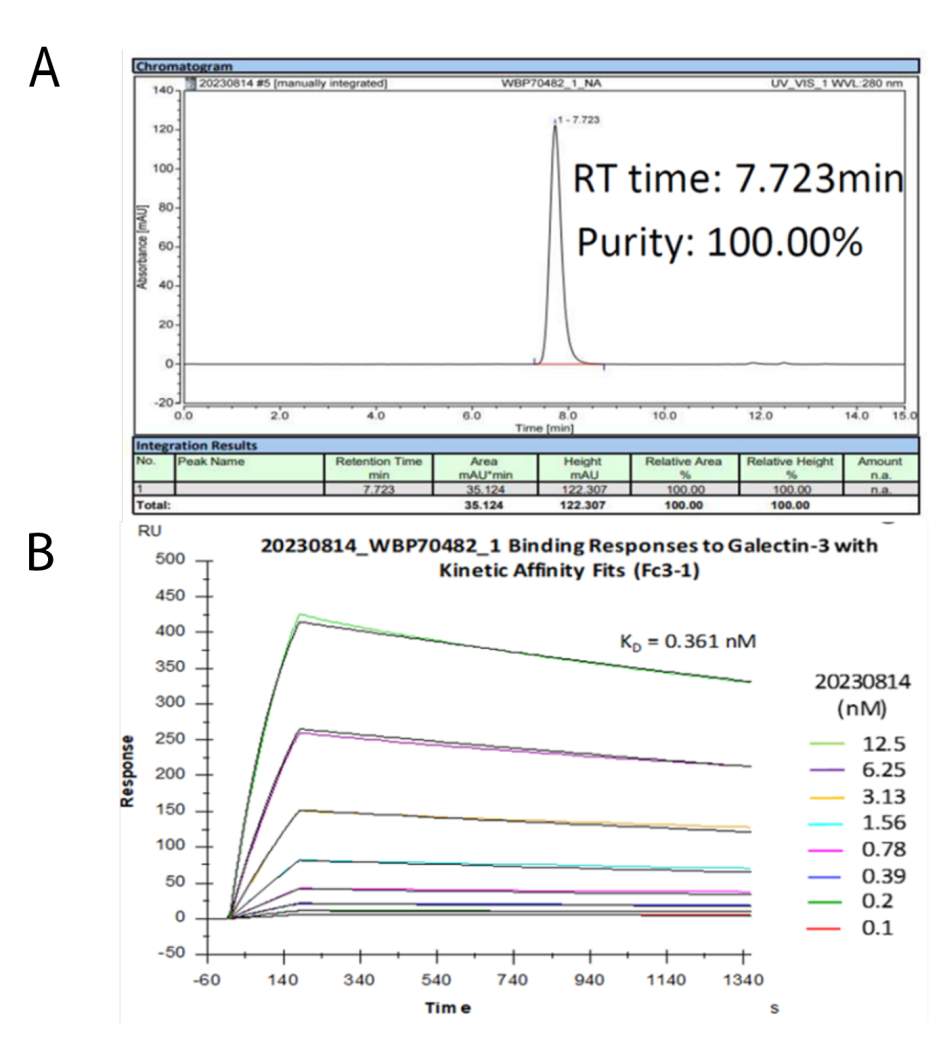


**Supplemental Figure S6.** Purity and binding activity of anti-Galectin-3 antibody. (A) SEC-HPLC chromatogram shows a single, symmetric peak with a retention time of 7.723 minutes and 100% purity, indicating the absence of aggregates or higher-order structures. (B) Biacore surface plasmon resonance analysis confirms high-affinity binding to Gal-3, with a dissociation constant (KD) of 0.361 nM.

**Antibody-conjugated agarose resin efficiency and selectivity-** To evaluate the conjugation efficacy of the antibody to the resin, the absorbance at 280 nm of the antibody solution was recorded before and after the coupling. Conjugation efficiency was > 99.0%. The capacity of the antibody-conjugated resin to bind Gal-3 was tested by incubating the resin with human plasma. The % removal of Gal-3 was compared with naked agarose resin to test its selectivity and specificity. Briefly, 250ul resin was washed x4 with PBS to replace the storage solution then 15mL of human plasma was incubated with the resin for 1 hour at 300C. Plasma was removed after 1 hour and the resin was washed twice with PBS. Gal-3 concentrations were determined using an ELISA kit (REF #: 12727 RUO Gal-3 Kit, BG Medicine, Inc.) from several fractions: plasma prior incubation with the resin, plasma after incubation with the resin and elution by 0.1 M Gly-HCl, pH 2.8. The samples were also analyzed by SDS-PAGE including from the resin at the Gal-3 elution step either by 0.1 M Gly-HCl at pH 2.8 followed by neutralization step (pH 7.5) with 2M Tris-HCl, pH 8.5 and by boiling the resin. Gal-3 depletion comparison between conjugated resin and naked resin is depicted in Supplemental Figure S2C.

**Gal-3 depletion efficiency and capacity evaluation**

The adsorption efficiency of the Gal-3 apheresis column for Gal-3 was optimized by evaluating bead size and antibody density on the conjugated beads. A 2mL mini-column packed with the Gal-3 antibody-conjugated beads were tested using 60 column volumes of human plasma in a closed loop system. The plasma was re-circulated twice at the flow rate of 3 mL/min providing a residence time of 40 seconds in the column. Plasma was applied to columns from a tank having a rotating magnetic bead and post-column effluent was recirculated to the plasma tank in a closed loop. Samples were periodically collected from the tank, as well as before and after the column, to measure the continuous Gal-3 depletion and the Gal-3 removal efficiency across the column.

The bead matrix conjugated with the Gal-3 apheresis column antibody used in our porcine study demonstrated optimal performance. We consistently achieved close to 100% Gal-3 depletion, with post-column Gal-3 levels dropping to undetectable amounts at a residence time of 40 seconds (Supplemental Figure S2A). There was no saturation or decline in depletion efficiency, even at a plasma-to-column volume ratio of 60:1 (equivalent to 3 L of plasma in the XGal-3 column). Subsequent tests in an open system simulated the clinical flow rate scenario with a residence time of 80 seconds (equivalent to plasma flow of 37.5 mLl/minute), using a high plasma-to-column volume ratio of 240:1 (equivalent to 12L of plasma with the 50 mLl XGal-3 column). The results demonstrated nearly 100% Gal-3 depletion, with no detectable levels of Gal-3 after the column (Supplemental Figure S2B). These findings indicate that the column has sufficient capacity for large-volume plasma processing without compromising its depletion efficiency.


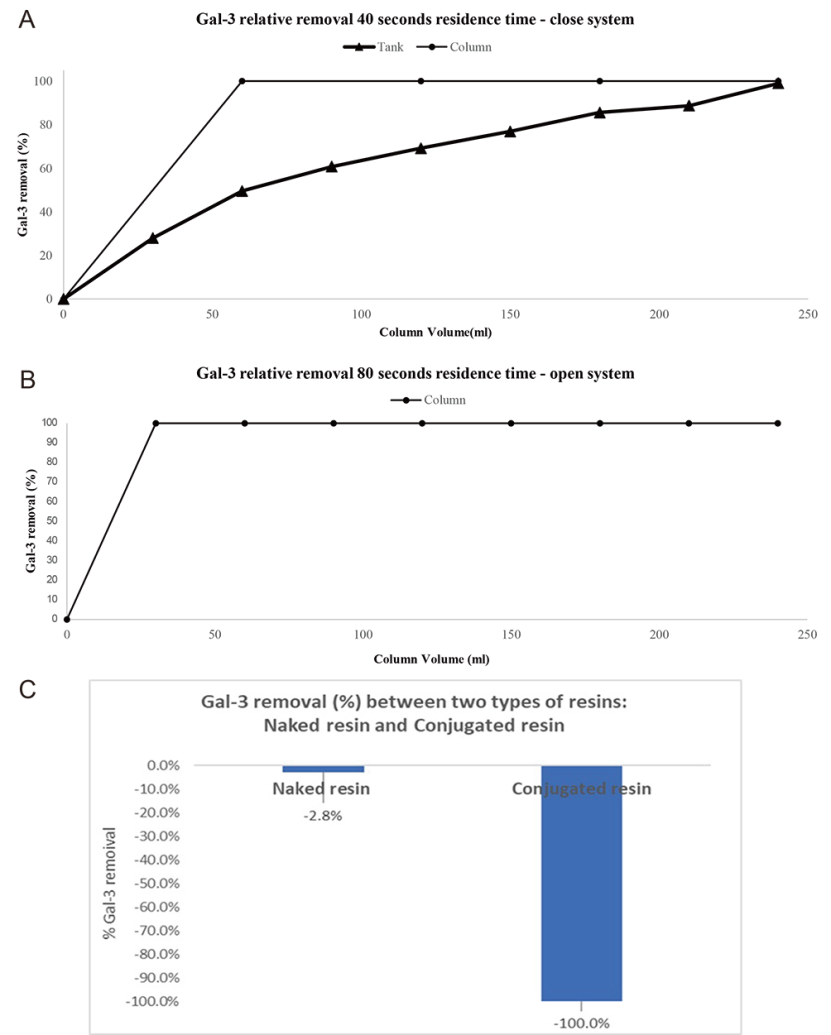


**Supplemental Figure S7**. Galectin-3 removal efficiency.

Following catheter placement surgery, a 60-minute period of stabilization commenced, after which baseline mean arterial pressure (MAP) was measured. LPS administration then began for 30 minutes, followed by a recovery period of 30 minutes, and then an acceleration period for 45 minutes. Gal-3 selective apheresis commenced 10 minutes into the acceleration period.

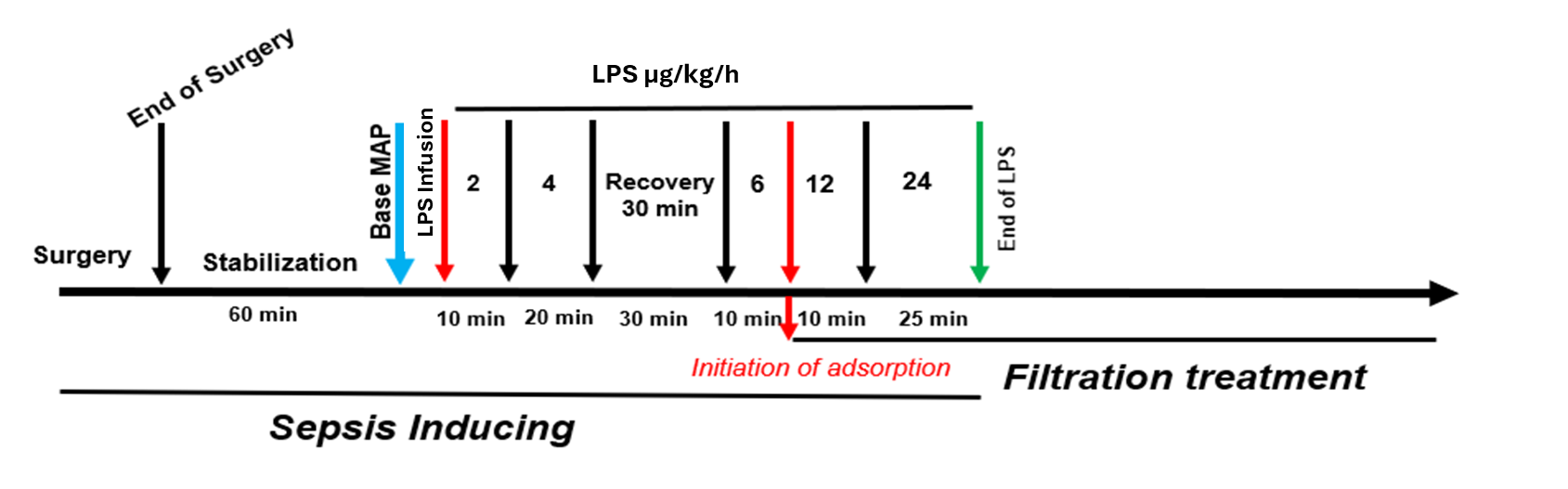
 **Supplemental Figure S8.** Timeline of porcine LPS-mediated sepsis induction followed by Gal-3 adsorption. (A) The percentage of Gal-3 removal as a function of column volume ratio processed (X-axis) at a residence time of 40 seconds in both the plasma tank (solid lines) and through the Gal-3 apheresis column (dashed lines). (B) The percentage of Gal-3 removal through the Gal-3 apheresis column as a function of plasma volume processed (X-axis) at a residence time of 80 seconds. (C) Comparative analysis of Gal-3 removal efficiency between naked resin and antibody-conjugated resin demonstrating the specificity of the conjugated system.

**Table S9**. Primer information

| Gene name | Primer sequence (5′–3′) |
| --- | --- |
| *CXCL2* | F-AAGTTTGTCTCAACCCCGCAG |
|  | R-TCTTCTTTCACTTTGATTCCGC |
| *IL-8* | F-AATACGCATTCCACACCTTTCC |
|  | R-CTGTTGTTGTTGCTTCTCAGTTCTC |
| *GAPDH* | F-GACATCAAGAAGGTGGTGAAGCA |
|  | R-GTCGTACCAGGAAATGAGCTTGA |
| *HIFA* | F-AGTGTACCTTAACTAGCAGGGGGAG |
|  | R-CAAATCAGCACCAAGCACG |
| *PIK3R1* | F-GCGCTGTACGACTATAAAAAGGAG |
|  | R-ATATATTCTACGTAAGTGCCTGGGA |
| *AKT* | F-GGCCCAACACCTTTGTCATAC |
|  | R-CTCACTGCCACTTCCATCTCCT |
| *VEGF* | F-CAACGACGAAGGTCTGGAGTGT |
|  | R-GCCTCGCTCTATCTTTCTTTGG |

**Table S10**. Antibodies used for immunostaining.

| Target | Application | Primary antibody (company) | Catalog No. | Dilution | Secondary antibody (company) | Catalog No. | Dilution |
| --- | --- | --- | --- | --- | --- | --- | --- |
| PI3K | IHC | Proteintech (PTG) | 60225-1-Ig | 1:400 | Abcam | AB6789 | 1:1000 |
| AKT | IHC | Proteintech (PTG) | 66444-1-Ig | 1:400 | Abcam | AB6789 | 1:1000 |
| HIF-1α | IHC | Boster | BS-0737R | 1:500 | Abcam | AB205718 | 1:2000 |
| vWF | IHC | Proteintech (PTG) | 27186-1-AP | 1:400 | Abcam | AB205718 | 1:2000 |
| CD31 | IHC | Abcam | ab182981 | 1:2000 | Abcam | AB205718 | 1:2000 |
| ZO-1 | IF | Abcam | ab221547 | 1:500 | Abcam | ab150078 | 1:400 |
| Occludin | IF | PTG | 27260-1-AP | 1:1000 | Abcam | ab150078 | 1:400 |
| NE | IF | Abclonal | A8953 | 1:100 | Abcam | ab150078 | 1:400 |
| MPO | IF | Abcam | ab208670 | 1:1000 | Abcam | ab150077 | 1:400 |
